# Evolutionary divergence of chloroplast FAD synthetase proteins

**DOI:** 10.1186/1471-2148-10-311

**Published:** 2010-10-18

**Authors:** Inmaculada Yruela, Sonia Arilla-Luna, Milagros Medina, Bruno Contreras-Moreira

**Affiliations:** 1 Estación Experimental de Aula Dei, Consejo Superior de Investigaciones Científicas (CSIC), Avda. Montañana, 1005, 50059 Zaragoza, Spain; 2 Institute of Biocomputation and Physics of Complex Systems (BIFI), Universidad de Zaragoza, Mariano Esquillor, Edificio I + D, 50018 Zaragoza, Spain; 3 Departamento de Bioquímica y Biología Molecular y Celular, Facultad de Ciencias, C/ Pedro Cerbuna 12, 5009 Zaragoza, Spain; 4 Fundación ARAID, Zaragoza, Spain

## Abstract

**Background:**

Flavin adenine dinucleotide synthetases (FADSs) - a group of bifunctional enzymes that carry out the dual functions of riboflavin phosphorylation to produce flavin mononucleotide (FMN) and its subsequent adenylation to generate FAD in most prokaryotes - were studied in plants in terms of sequence, structure and evolutionary history.

**Results:**

Using a variety of bioinformatics methods we have found that FADS enzymes localized to the chloroplasts, which we term as plant-like FADS proteins, are distributed across a variety of green plant lineages and constitute a divergent protein family clearly of cyanobacterial origin. The *C*-terminal module of these enzymes does not contain the typical riboflavin kinase active site sequence, while the *N*-terminal module is broadly conserved. These results agree with a previous work reported by Sandoval *et al*. in 2008. Furthermore, our observations and preliminary experimental results indicate that the *C-*terminus of plant-like FADS proteins may contain a catalytic activity, but different to that of their prokaryotic counterparts. In fact, homology models predict that plant-specific conserved residues constitute a distinct active site in the *C*-terminus.

**Conclusions:**

A structure-based sequence alignment and an in-depth evolutionary survey of FADS proteins, thought to be crucial in plant metabolism, are reported, which will be essential for the correct annotation of plant genomes and further structural and functional studies. This work is a contribution to our understanding of the evolutionary history of plant-like FADS enzymes, which constitute a new family of FADS proteins whose *C*-terminal module might be involved in a distinct catalytic activity.

## Background

Flavin mononucleotide (FMN) and flavin adenine dinucleotide (FAD) are essential cofactors for numerous enzymes (*i.e.*, dehydrogenases, oxidases, reductases) that participate in one- and two-electron oxidation-reduction processes critical to the major metabolic routes in all living organisms [1-4]. Riboflavin (RF), the precursor of FMN and FAD can be *de novo *synthesized by plants, fungi and many bacteria, but in mammals the only known RF source is the exogenous riboflavin (vitamin B_2_) obtained through the diet [5-7].

In most prokaryotes, the synthesis of FMN and FAD is catalyzed from RF and ATP by a single bifunctional enzyme, usually known as FAD-synthetase (FADS), through the sequential action of its two enzymatic activities: an ATP:riboflavin kinase (RFK, EC 2.7.1.26) that transforms RF and ATP into FMN, and an ATP:FMN adenylyltransferase (FMNAT, 2.7.7.2) that catalyzes the subsequent adenylylation of FMN to FAD. Thus, FADS is a bifunctional RFK/FMNAT enzyme [8]. FADSs are typically 310-340 residues in length and are folded in two modules [9-11], each one mainly involved in one of the activities. The RFK reaction has been related with the *C*-terminal module (RFK-module), while the *N*-terminal module is mainly related to the FMNAT activity (FMNAT-module); hence, two independent substrate binding and catalytic sites are in charge of each activity [11,12]. In one hand, the RFK-module (~ 180 aa) folds in a globular domain and its overall topology is similar to that found in the RFKs from *Homo sapiens *(*Hs*RFK) and *Schizosaccharomyzes pombe *(*Sp*RFK), with differences only observed in the loops connecting secondary structure elements [13,14]. Furthermore, the substrate binding motifs PTAN and GxY of the RFK-module are conserved among FADSs and eukaryotic RFKs. In the other hand, the FMNAT-module consists of an α/β dinucleotide binding domain with a typical Rossmann fold topology (~ 150 aa) [9-11]. Moreover, it seems to be remotely similar to the nucleotidyltransferase (NT) superfamily and contains the typical (H/T)xGH and xSST/SxxR motifs involved in binding nucleotide and phosphate groups. Interestingly, monofunctional enzymes with only RFK activity have been described in *Bacillus subtilis *[15] and *Streptococcus agalactiae *[16] but no monofunctional FMNAT enzymes have been reported in prokaryotes.

A different scenario is found in eukaryotes, where both activities are generally split in two different enzymes with either RFK or FMNAT activity [17-20]. As mentioned above, the RFK enzymes show sequence and structure similarity to the RFK-module of prokaryotic FADS [13,14]. However, eukaryotic FMNATs share little or no sequence similarity to the FMNAT-module of FADS, as these enzymes belong to two different protein superfamilies, which are thought to require different sets of active-site residues to carry out the same chemistry [21-23]. The eukaryotic FMNAT-module is currently classified as a member of the 3'-phosphoadenosine 5'-phosphosulfate (PAPS) reductase-like family belonging to the "adenosine nucleotide α-hydrolase-like" superfamily, which has motifs different from those of NTs.

In plants only a few efforts have been devoted to this system. Early studies characterized apparently monofunctional enzymes with either RFK or FMNAT activities in several plant species [24-27]. In those studies the subcellular localization of RFK and FADS was not addressed although it is known that plants use flavin nucleotides in mitochondria, plastids and in the cytosol. In an earlier work RFK activity was associated to the cytosol and to an organellar fraction containing chloroplasts and mitochondria [28]. More recently, a bifunctional enzyme with both FMN hydrolase and RFK activities has been described in *Arabidopsis thaliana *(*At*FHy/RFK) [29], whose *N*-terminal module responsible for the FMN hydrolase activity, shares sequence similarity with members of the haloacid dehalogenase (HAD) superfamily. *At*FHy/RFK enzyme was predicted to be cytosolic [29]. Additionally, two more enzymes with FMNAT activity have been identified, cloned and characterized in the same species [30]. These *At*RibF1 and *At*RibF2 enzymes, herein plant-like FADS proteins, have an *N*-terminal module which is found to be homologous to the FMNAT-module of FADS, but instead its *C*-terminal module does not catalyze RF phosphorylation. *At*RibF1 and *At*RibF2 were localized to the chloroplast [30]. In mitochondria, the catalytically conversion of RF into FMN and FAD has been reported, due to the existence of mitochondrial RFK and FADS enzymes [31], but nevertheless FADS activity was much lower than in chloroplasts. These results agree with the cited confocal microscopy studies [30], but the hypothesis for the localization of FADS isoforms (*At*RibF1 and *At*RibF2) in mitochondria cannot be ruled out on the basis of bioinformatics (TAIR) analysis [31]. The mitochondrial FAD-forming enzymes reside in two distinct monofunctional enzymes, which can be separated in soluble and membrane-enriched fractions. It is worth mentioning that the genes encoding organellar RFK activity remains unidentified.

In order to investigate RFK and FMNAT activities in plants we have conducted an extensive bioinformatics survey using the available genomes in public databases. Here we report the identification of a conserved *C*-terminal module in plant FADS enzymes, which does not contain the typical RFK active site sequence, suggesting that it belongs to a new family of FADS proteins. The activity of this module is discussed.

## Results and Discussion

### Sequence and evolutionary analysis

**Figure 1:**
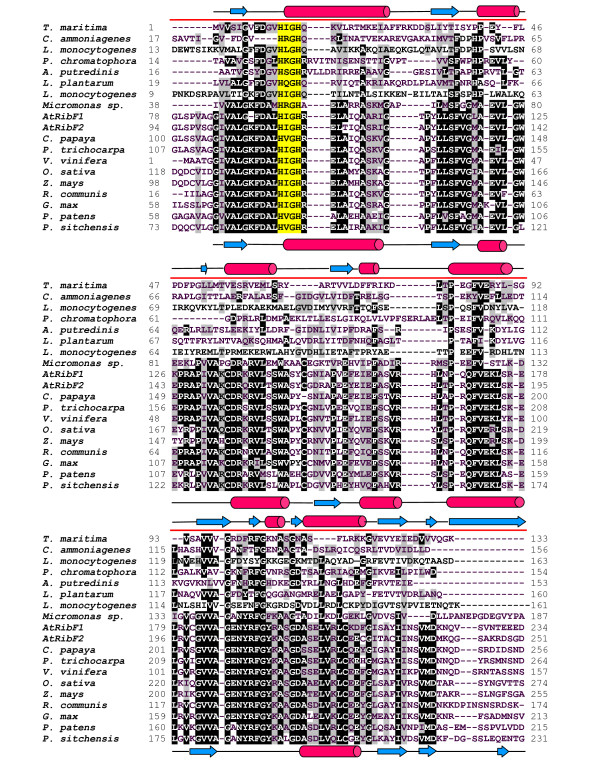
**Alignment of a selection of FADS protein sequences used in this study (part 1 of 2)**. The alignment includes bifunctional FADS-type I proteins from *Thermotoga maritima *(Q9WZW1), *Corynebacterium ammoniagenes *(Q59263), *Listeria monocytogenes *HCC23 (YP_002350202) and *Paulinella chromatophora *(YP_002048796.1), FADS-type II proteins from *Alistipes putredinis *DSM 17216 (ZP_02425815.1), *Lactobacillus plantarum *JDM1 (YP_003062293.1) and *Listeria monocytogenes *HCC23 (YP_002350850), and plant-like FADS from *Micromonas *sp. RCC299 (XP_002501784), *Arabidopsis thaliana *L., *At*RibF1 (At5g23330) and *At*RibF2 (At5g08340), *Carica papaya *(Cp00060g00820), *Populus trichocarpa *(Pt07g06690, EEE90505.1), *Vitis vinifera *L. (Vv00g06080, XP_002273393.1), *Oryza sativa *L. (Os03g58710, NP_001051594), *Zea mays *L. (NP_001151161.1), *Ricinus communis *L. (XP_002517319.1), *Glycine max *(EST assembly from Soybean Genome Project, DoE Joint Genome Institute), *Physcomitrella patens *L. (Pp00229g00440, A9TH63) and *Picea sitchensis *L. (ABR16575.1). *N*-terminus (red) and *C*-terminus (green) within FADS proteins are marked over the sequences. Secondary structure of *Tm*FADS (pdb 1mrz) is shown in the upper line. Predicted secondary structure of *At*RibF1 is shown in the bottom line. Conserved amino acids are shown in black. Catalytic motifs in FADS enzymes are highlighted in yellow.

**Figure 2:**
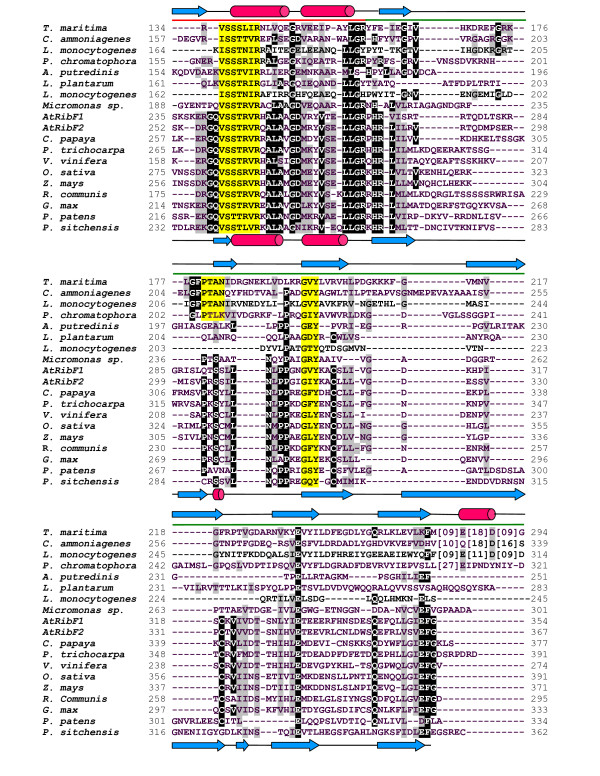
**Alignment of a selection of FADS protein sequences used in this study (part 2 of 2)**. The alignment  includes bifunctional FADS-type I proteins from *Thermotoga maritima *(Q9WZW1), *Corynebacterium ammoniagenes *(Q59263), *Listeria monocytogenes *HCC23 (YP_002350202) and *Paulinella chromatophora *(YP_002048796.1), FADS-type II proteins from *Alistipes putredinis *DSM 17216 (ZP_02425815.1), *Lactobacillus plantarum *JDM1 (YP_003062293.1) and *Listeria monocytogenes *HCC23 (YP_002350850), and plant-like FADS from *Micromonas *sp. RCC299 (XP_002501784), *Arabidopsis thaliana *L., *At*RibF1 (At5g23330) and *At*RibF2 (At5g08340), *Carica papaya *(Cp00060g00820), *Populus trichocarpa *(Pt07g06690, EEE90505.1), *Vitis vinifera *L. (Vv00g06080, XP_002273393.1), *Oryza sativa *L. (Os03g58710, NP_001051594), *Zea mays *L. (NP_001151161.1), *Ricinus communis *L. (XP_002517319.1), *Glycine max *(EST assembly from Soybean Genome Project, DoE Joint Genome Institute), *Physcomitrella patens *L. (Pp00229g00440, A9TH63) and *Picea sitchensis *L. (ABR16575.1). *N*-terminus (red) and *C*-terminus (green) within FADS proteins are marked over the sequences. Secondary structure of *Tm*FADS (pdb 1mrz) is shown in the upper line. Predicted secondary structure of *At*RibF1 is shown in the bottom line. Conserved amino acids are shown in black. Catalytic motifs in FADS enzymes are highlighted in yellow.

As shown in Table 1, most prokaryotic genomes (1178/1194) surveyed in this study, including cyanobacteria, contain a single gene encoding for a bifunctional FADS enzyme, hereafter identified as FADS-type I protein. Sequence searches in a variety of repositories of green plant sequences allowed us to identify a related group of genes, which contain two domains with high similarity (see Methods section) to FADS-type I sequences (Figure 1 and 2), and are also present in a single copy in most cases with currently available complete genome (14/18). This result agrees with previous work by Sandoval *et al*. [30]. The *N*-terminal module of these proteins displays high similarity to the FMNAT-module of prokaryotic FADS-type I, showing the typical motifs HxGH and xSST/SxxR involved in FMNAT activity, also common to other NTs. However, several observations can be made with respect to the *C*-terminal module in plant proteins: *i) *its length is 40 to 60 residues shorter; *ii) *the PTAN motif characteristic of the RFK activity mutates to PxS; *iii*) a LNxPP motif is found conserved in plants, next to the invariant GxY motif. *At*RibF1 and *At*RibF2 belong to this group of proteins and were recently characterized by Sandoval *et al*. [30], who did not detect any RFK activity in these enzymes. This experimental observation, together with the absence of the PTAN motif suggests a different enzymatic activity for this module. Therefore we named these proteins as plant-like FADS.

**Table 1:** Bacterial genomes containing FADS-like proteins ^(1)^

Type of protein	Genomes	N
FMNAT RFK	*Chthoniobacter flavus *Ellin428 ctg76 *Mesoplasma florum *L1 *Mycoplasma capricolum subsp. capricolum* *Mycoplasma mycoides subsp. capricolum*	4

FADS-type I	Most bacterial genomes	1171

FADS-type I FMNAT	*Bacillus cereus *03BB102, *Bacillus cereus *ATCC 10987 *Bacillus turingiensis str. AlHakam* *Geobacillus thermodenitrificans *NG80-2 *Listeria monocytogenes *EGD-e	5

FADS-type I RFK	*Bacillus subtilis subsp. subtilis* *Haemophilus influenzae *86-028NP	2

FADS-type I FADS-type II	*Arthrobacter chlorophenolicus *A6 *Bacillus cereus subsp. cytotoxis *NVH 391-98 *Lactobacillus plantarum *JDM1 *Lactobacillus plantarum *WCFS1 *Listeria monocytogenes *HCC23 *Listeria welshimeri serovar* *Oceanobacillus iheyensis *HTE831 *Treponema denticola *ATCC 35405	8

FADS-type II	*Eubacterium saphenum *ATCC 49989 *Mycoplasma conjunctivae* *Treponema pallidum subsp. pallidum str. Nichols*	3

FADS-type II RFK	*Alistipes putredinis *DSM 17216	1

^1 ^Check details in section "Sequence analysis" of Methods.

Furthermore, a few bacterial parasites and pathogens isolated from plant, human or soil material and belonging to phyla *Firmicutes*, *Actinobacteria, Tenericutes and Spirochaetes *contain extra sequences with significant similarity to FADS-type I (E-values ≤ 1.5×10^-10^). However, as shown in Figure 1 and 2, these sequences do not conserve the catalytic PTAN motifs, and have shorter *C*-terminal modules similar in length to plant like-FADS, suggesting that they might constitute another divergent type of FADS, which we label as FADS-type II (see Table 1).

In our sequence searches, plant-like FADS proteins are distributed across a variety of green plant lineages. Among land plants we found 84 matches (62 in Eudicots, 11 in Monocots, 6 in Coniferophyta, 3 in Magnoliids, 1 in Bryophyta, 1 in Lycopodiophyta). Other than land plants, plant-like FADS proteins were restricted to unicellular photosynthetic organisms belonging to phylum *Chlorophyta *(*Micromonas pusilla, Chlamydomonas reinhardtii, Coccomyxa *sp. and *Ostreococcus lucimarinus*) (see Table 2). All plant genomes surveyed encode these proteins in the nuclear genome (sequence searches in chloroplast genomes did not produce matches). Our search strategies did not find plant-like FADS proteins in any other eukaryotic genomes. These observations might imply that these genes have a prokaryotic origin, somewhat related to the endosymbiotic origin of chloroplasts. On the other hand, all green plant genomes explored have a copy of the cytosolic bifunctional FHy/RFK protein except two *Micromonas *species that have a monofunctional RFK enzyme like in most eukaryotes. Proteins related to the HAD domain of FHy/RFK have been found in either bacteria or eukaryotes but this enzyme has been suggested to be unique in plant lineages probably being originated by fusion of a HAD to an eukaryotic-type RFK [29].

**Table 2:** Eukaryotic genomes containing FADS-like proteins ^(1)^

Type of protein	Genomes	N
FADS-type I	*Anopheles gambiae* *Babesia bovis* *Caenorhabditis japonica *strain DF5081 *Caernorhabditis remanei *strain PB4641 *Culex pipiens quienquefasciatus* *Plasmodium knowlesi* *Trichoplax adhaerens* *Paulinella chromatophora*	8

RFK	Most eukaryotic genomes	658

Plant-like FADS RFK	*Micromonas pusilla* *Micromonas *sp. RCC299	2

Plant-like FADS FHy/RFK ^(2)^	Land plant genomes *Chlamydomonas reinhardtii* *Coccomyxa sp*. *Ostreococcus lucimarinus*	87 ^(3)^

^1 ^Check details in section "Sequence analysis" of Methods.

^2 ^FMN hydrolase fused to RFK in ref. 29

^3 ^Includes unigenes compiled from ESTs.

**Figure 3:**
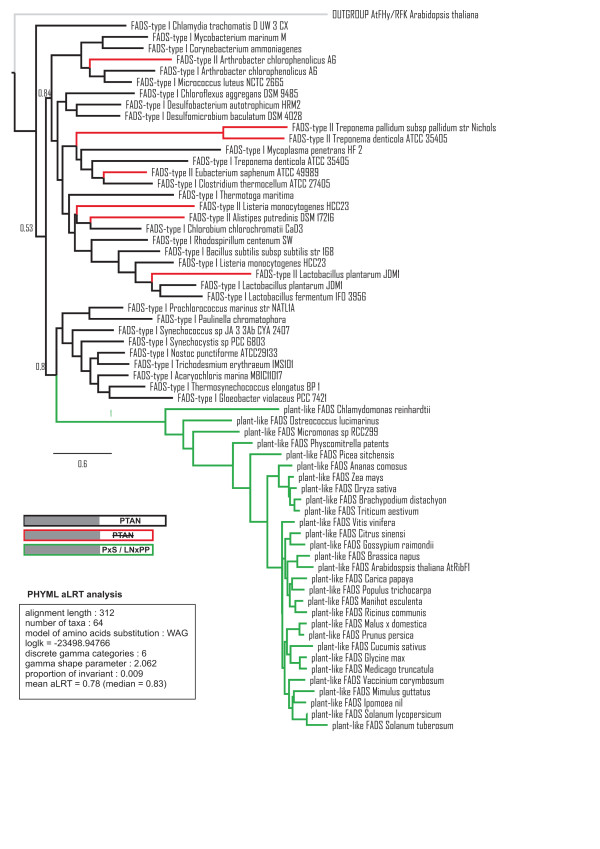
**Maximum likelihood tree of a selection of 64 FADS sequences**. Maximum likelihood tree of a selection of 64 sequences, including FADS-type I (black), FADS-type II (red) and plant-like FADS (green) proteins. Approximate likelihood ratio support values are printed next to branches and a summary of the tree parameters is shown in a box. Schematic protein representations including both the *N*-terminal (in grey) and the *C*-terminal modules, highlighting the distinctive sequence motifs, are drawn to assist in the interpretation of the tree. The underlying multiple alignment is available in Additional file 1; Figure S3.

With the aim of further exploring the origin of plant-like FADS proteins, we carried out a phylogenetic analysis, which is summarized in the phylogram in Figure 3. According to this tree, which was rooted by taking the sequence of the cytosolic protein *At*FHy/RFK from *Arabidopsis thaliana *as an outgroup, plant-like FADS proteins are closer to the group of cyanobacteria than any other bacterial species, which were selected to represent taxa included in Table 1. Indeed proteins from both cyanobacteria and green plants are enclosed in a clade with an associated approximate likelihood ratio (aLRT) of 0.80. These observations suggest that plant-like FADS proteins have a prokaryotic origin closely related with cyanobacteria, although shaping a divergent group of sequences, as illustrated in Figure 3. Note that *At*FHy/RFK clusters apart from prokaryotic FADS, confirming a different origin for this enzyme. The tree also suggests that plant-like FADS proteins diverged from bacterial FADS probably before the separation of the two major plant phyla (*Streptophyta -*plants and their closest green algal relatives- and *Chlorophyta *-the rest of green algae-), since they are present in species from both. In order to further investigate this, we searched for putative plant-like FADS homologues in *Mesostigma viride*, proposed to be the earliest plant lineage and anterior to the divergence of the *Streptophyta *and *Chlorophyta *[32]. Unfortunately, the nuclear genome of this species is not available and the chloroplast and mitochondrial genomes yielded no sequence matches. Our findings could indicate that plant-like FADS indeed derived from cyanobacterial FADS, despite the fact that they are now encoded in the nuclear genome [33].

Moreover, these results reveal that most bacteria containing FADS-type II sequences have also typical FADS-type I proteins (see Table 1) and the tree in Figure 3 shows that these two types of sequences cluster together, implying that they might actually be paralogous genes. Only the genomes of *Eubacterium saphenum *ATCC 49989, *Mycoplasma conjunctivae, Treponema pallidum subsp. Pallidum *contain exclusively FADS-type II proteins. Although the tree does not support that FADS-type II proteins constitute a distinct evolutionary class, their shorter and non-conserved *C*-terminal domains still clusters them clearly as a distinct functional group, which might have lost the *C*-terminal activity typical of FADS-type I proteins.

We also note the observed variability in terms of RFK and FMNAT enzymatic activities in bacterial genomes. While most prokaryotes have a single copy of a typical FADS-type I sequence, in 4 species both enzymatic activities are separated in monofunctional proteins, which correspond to RFK or FMNAT modules, respectively. In other cases the FADS-type I sequence was accompanied by either monofunctional prokaryotic FMNAT (5 genomes) or monofunctional prokaryotic RFK (2 genomes). For instance, the genome of *Alistipes putredinis *contains both a monofunctional RFK and a FADS-type II sequence. Furthermore, although most bacterial FADS and RFK proteins include the conserved PTAN motif, some sequence variants can be found, including PTLK, PTLN, PTIN or KTAN, which nevertheless conserve the *C*-terminal module length. As these genomes do not contain any other RFK related proteins, these sequence variants are supposed still to be responsible for the RFK activity.

FADS-type I sequences were also found in 8 eukaryotic species (Table 2), including *Anopheles gambiae*, *Caenorhabditis *sp., *Trichoplax adhaerens*, which is considered to be the most primitive multi-cellular animal known, or the freshwater amoeba *Paulinella chromatophora*, which harbours a cyanobacterial endosymbiont.

It has been proposed that the double enzymatic activity of FADS proteins might be the result of a gene fusion event that genetically perpetuated an ancient protein-protein interaction [8,34]. If this hypothesis holds true, it is remarkable that 1190 out of 1194 bacterial genomes have a copy of this fused gene (Table 1) while monofunctional RFKs are vastly predominant (658/755) across eukaryotic genomes (Table 2). This observed unbalance suggests that this fusion event, or functional coupling, would have been evolutionary favoured only in unicellular organisms, from which chloroplasts are thought to be derived.

We would like to remark that FADS proteins are annotated in sequence databases with confusing or contradictory names such as riboflavin biosynthesis protein RibF (*i.e*., YP_002487514.1), FMN adenylylate transferase (*i.e*., NP_692523.1), FMN adenylyltransferase (*i.e.*, YP_001623829.1), FAD synthase (*i.e*., YP_518746), riboflavin kinase/FMN adenylyltransferase (*i.e*., YP_932710), flavokinase/FAD synthetase (*i.e*., YP_002783884), riboflavin kinase/FAD synthetase (*i.e*., NP_975116.1). Indeed, non-strictly FADS sequences are also named as that (*i.e.*, YP_003062293.1). In the case of plants, plant-like FADS sequences are found as riboflavin kinase (*i.e.*, gb|CO899788.1|, gb|BG509026.1|, gb|CN491424.1|) or protein-s isoprenylcysteine o-methyltransferase (*i.e.*, PTHR12714, gb|GR935784.1)|, cassava1385). This misleading variability in names is of no benefit to users, and clearly so a consensus in the nomenclature would be desirable. We hope this work makes a contribution in this direction.

### A putative molecular function for the C-terminal module in plant-like FADS proteins

PSI-BLAST searches of both the complete sequence of the plant-like FADS *At*RibF1 and its *C-*terminus matched only NTs and RFKs (10 iterations, E-value < 3×10^-8^) from bacteria, cyanobacteria, yeast and human. No other family was identified as related to the *C*-terminus of plant-like FADS. The similarity between the newly identified *C*-terminal module and NTs was further explored in the pdb70 structural library using the fold-recognition algorithm HHPred in local and global mode. Local searches provided significant matches (E-value ≤ 1.4×10^-17^) to: the RFK-module of *Tm*FADS (pdb 1mrz, 1s4m, 1t6x, 1t6y, 1t6z, 2i1l; [9,10]), *Sp*RFK (pdb 1n08, 1n05, 1n07, 1n06; [13]), *Hs*RFK (pdb 1nb0, 1nb9, 1p4m, 1q9s; [14]) and *Trypanosoma brucei *RFK (pdb 3bnw). Apart from these hits, global searches with the *C-*terminal domain yielded significant matches (E-values ≤ 2.5×10^-6^) to: nicotinamide mononucleotide (NMN) adenylyl transferase/ribosylnicotinamide kinase from *Haemophilus influenzae *(pdb 1lw7), ethanolamine-phosphate cytidylyltransferase from *H. sapiens *(pdb 3elb), nicotinamide-nucleotide adenylyltransferase (pdb 2qjt) from *Francisella tularensis *and the *C*-terminal module of bifunctional nicotinamide mononucleotide (NMN) adenylyltransferase/Nudix hydrolase from *Synechocystis *sp. (*Sy*NadM-Nudix) (pdb 2qjo; [35]).

**Figure 4:**
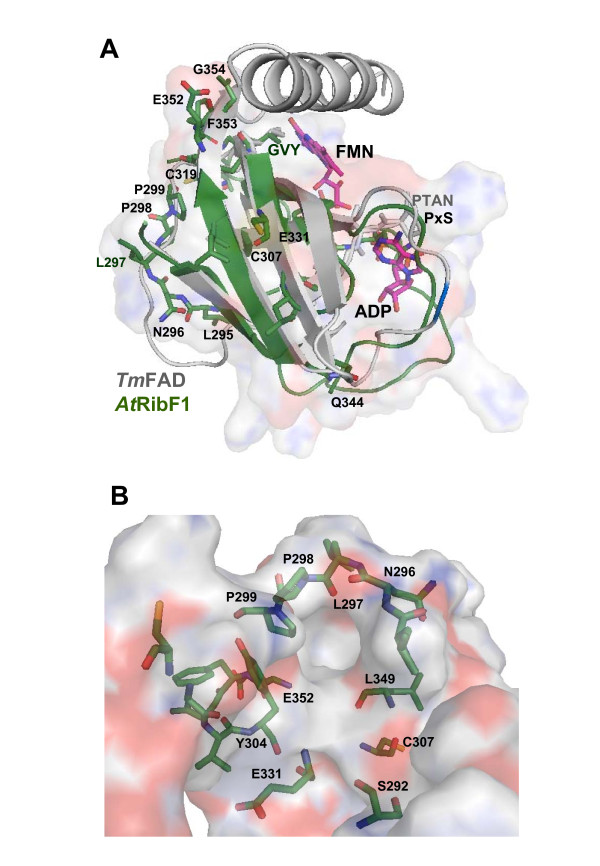
**Structural comparison of the *C*-terminal module of both *Tm*FADS (pdb **1t6y**) and the homology model of *At*RibF1**. A) Ribbon diagrams of *Tm*FADS (gray) and homology model of *At*RibF1 (green). Bound ADP and FMN ligands in pdb 1t6y are shown as sticks in CPK with carbons in magenta. Conserved residues in the *C*-terminus of plant-like FADS are superposed to catalytic residues in *Tm*FADS. Electrostatic potential surface of *At*RibF1 is also shown. B) Putative new active site in the structural model of *At*RibF1. Specific-plant conserved residues are shown as sticks with carbons in green.

To evaluate the possible role of the *C*-terminal module of plant-like FADSs and the molecular arrangement of this protein region we modelled the *At*RibF1 structure using all the above mentioned protein structures as templates. The best predicted models, as expected in terms of sequence similarity and alignment quality in putative binding and catalytic regions were obtained with the X-ray structure of *Tm*FADS, *Sp*RFK and *Hs*RFK. In order to annotate putative functional residues, the comparative models were superposed to the original templates, including all ligands present in the experimental coordinates. The superposition in Figure 4 indicate that plant conserved residues ^290^P×S^292 ^and ^302^GVY^304 ^in the model occupy equivalent positions with respect to the ligands (ADP and FMN) present in the crystallographic structure of *Tm*FADS. Similar results were obtained with *Sp*RFK and *Hs*RFK (data not shown). The predicted secondary structure of *At*RibF1 corresponds well with that of *Tm*FADS (see Figure 1 and 2), although it lacks the last α-helix at the *N*-terminal end, which is conserved also in *Sp*RFK and *Hs*RFK. This helix appears to be crucial for a correct orientation of the bound flavin substrate [14] and its absence in *At*RibF1 is in agreement with the observed lack of RFK activity [30].

Figure 4 shows the specific-plant conserved residues Leu295, Asn296, Leu297, Pro298 and Pro299 (^295^LNLPP^299 ^motif), Cys307, Cys319, Glu331, Gln344, Glu352, Phe353 and Gly354. It can be observed that the LNLPP motif is located in a flexible loop, in an opposite site of that bound FMN or ADP in *Tm*FADS and orientated towards a cavity. Furthermore, the conserved residues Ser292, Cys307, Glu331 and Glu352 appear orientated towards this cavity suggesting that this site could possibly be a putative new binding-site in plant-like FADSs. It is also worth mentioning that Glu331 residue, invariant also in FADS and RFK families (*i.e.*, Glu268 in FADS-type I from *Corynebacterium ammoniagenes *[11]), has been proposed to act as a catalytic base.

As mentioned above, remote similarity of the *C*-terminal module of plant-like FADSs was found with the *C-*terminal domain of other families such as *Sy*NadM-Nudix (E-value = 6.8×10^-08^), which belongs to a large superfamily of pyrophosphohydrolases (see Additional file 1; Figure S4). In *Arabidopsis *27 Nudix hydrolase genes have been found and the proteins they encode are able to hydrolyze various types of nucleoside diphosphates derivatives such as ADP-glucose, ADP-ribose and a wide range of its derivatives, FAD, NADH, NADPH, and diadenosine polyphosphates [36]. Moreover, a remote sequence consensus of this protein region including the LNxPP motif was found with serine/threonine phosphatases 2C and members of the hydrolase superfamily. These observations suggest that the *C*-terminal module of *At*RibF1 could have a function other than RFK enzymatic activity. Sandoval *et al*. [30] showed that purified recombinant *At*RibF1 and *At*RibF2 enzymes only display FADS activity, with undetectable RFK activity and hence assumed that these are indeed monofunctional enzymes. However, they were able to measure FMN hydrolase, FAD pyrophosphatase and RFK activities in Percoll-isolated chloroplasts.

As mentioned above, our bioinformatic analyses point out that plant-like FADS proteins could be bifunctional enzymes. More precisely, structural similarities predict a hydrolase and phosphatase activity for the *C*-terminal module, although the possibility to have a non-enzymatic regulatory role or to be a simple evolutionary relic should not be dismissed. Nevertheless, considering the results of Sandoval *et al. *[30] and ours, we could speculate that some of the measured activities in isolated chloroplasts (*i.e*., FMN hydrolase or FAD pyrophosphatase) could be associated to this *C*-terminal module. In order to test this hypothesis we have designed some experiments with recombinant plant-like FADS from soybean (*Glycine max*) and preliminary results seem to indicate that its *C*-terminal module might have a hydrolytic activity since *Gm*FADS was able to convert FMN into RF (data not shown). Interestingly, this activity was not detected in purified FADS from *C. ammoniagenes*, a typical FADS-type I protein [11]. While these preliminary results seem to be in agreement with our theoretical analyses, clearly further investigations are necessary to confirm the possible enzymatic role of the *C*-terminal module of plant-like FADS. Future work will be done by using recombinant plant-like FADS (*Gm*FADS) in order to confirm this observed hydrolytic enzymatic activity.

## Conclusions

Plant-like FADS enzymes are distributed across a variety of green plant lineages and constitute a divergent protein family clearly of cyanobacterial origin. Homology models predict that plant-specific conserved residues are orientated towards a cavity, building a distinct active site when compared to that involved in substrate binding and catalysis in the *C*-terminus of typical FADS-type I enzymes. The remote relationship reported here between plant-like FADS proteins and members of pyrophosphohydrolase or phosphatase superfamilies as well as preliminary experimental results suggest that the *C*-terminal module of these proteins, clearly of bacterial origin, might be involved in a catalytic function.

## Methods

### Sequence analysis

The NCBI non-redundant protein sequences (nr), nucleotide collection (nr/nt) and concise microbial protein databases, and the CyanoBase (http://genome.kazusa.or.jp/cyanobase/) sequence library, were scanned with PSI-BLAST [37] and TBLASTN, in order to retrieve sequences similar (E-values < 10^-14^) to: *i) *FADS from *Thermotoga maritima *(UniProtKB Q9WZW1 [9,10]), *ii) *RFK from *Bacillus subtilis *(GenBank AAC00333.1) and *iii) *the plant-like FADS *At*RibF1 (At5g23330, NP_568429, GenBank ACH56223.1) or *At*RibF2 (At5g08340; NP_568192, GenBank ACH56224.1). To increase sensitivity, further similar sequences were retrieved by scanning either the *N-*terminal or the *C*-terminal modules of prokaryotic and plant-like FADS and RFK proteins.

In order to increase the recovery of plant sequences, which are currently distributed from a variety of repositories, the *At*RibF1 sequence was also scanned against JGI Genome portal (http://genome.jgi-psf.org/), Phytozome (http://www.phytozome.net/) and PLAZA (http://bioinformatics.psb.ugent.be/plaza/) with E-values < 10^-50^. Finally, further searches were performed against NCBI Expressed Sequence Tags (filter: *Viridiplantae*) and TIGR Plant Transcript Assembly databases, with E-values < 10^-20^.

### Multiple alignments

The multiple alignment in Figure 1 and 2 was constructed in three steps, using the *At*RibF1 protein as seed:

1) A sequence profile of plant-like proteins was compiled with ClustalW [38].

2) A selection of bacterial and eukaryotic sequences was aligned to the profile.

3) The sequence of *Thermotoga maritima *was added following the fold recognition alignment produced by HHPred [39] using the Protein data Bank structure 1mrz. This template was predicted to be the best modelling template by the BioInfoBank Meta Server (see below).

The multiple alignment used to drive the phylogenetic analysis summarized in Figure 3 was constructed as follows:

1) A representative set of FADS-type I and FADS-type II sequences were multiply aligned with CLUSTALW [38] and their secondary structure was predicted with PSIPRED [40] taking the *Thermotoga maritima *sequence as a representative. The sequences selected are representative of bacterial species having FADS-type I and/or FADS-type II, and belonging to phyla *Actinobacteria, Firmicutes*, *Spirochaetes and Tenericutes*. Also sequences from species containing only FADS-type I, which belong to phyla *Chlamydiae*, *Chlorobi, Chloroflexi *(green non-sulfur bacteria), *Cyanobacteria, Proteobacteria *(purple bacteria) and *Thermotogae *are included, providing a good coverage of diverse phylogenetic bacterial groups.

2) The sequence of the cytosolic protein *At*FHy/RFK from *Arabidopsis thaliana *[29] was added and aligned as an outgroup, and the resulting multiple alignment was converted to a hidden Markov model in HHSearch format with *hhmake *[39].

3) All plant-like FADS protein sequences that covered most of both domains (from the HxGH to the GxY motif) were considered complete, aligned with CLUSTALW [38] and converted to a hidden Markov model, including the PSIPRED secondary structure prediction of *At*RibF1. The plant sequences selected cover the diverse phylogenetic groups of green plants as shown in Additional file 1; Figure S1.

4) The profiles 2) and 3) were globally aligned with *hhalign *[39] and the resulting alignment was trimmed by removing the poorly aligned segments, following the protocol "automated1" of the trimAL software (http://trimal.cgenomics.org/) [41]. The original and trimmed alignments are available in Additional file 1; Figures S2 and S3.

### Phylogenetic analysis

The trimmed multiple alignment described above was used to drive a maximum likelihood phylogenetic tree with PhyML [42] and the best fitting amino acid substitution model selected with ProtTest [43]. The tree was midpoint-rooted and plotted with FigTree (http://tree.bio.ed.ac.uk/software/figtree).

### Molecular modelling

To further investigate possible molecular functions of the *C*-terminal module of plant-like FADS proteins the complete protein sequence of *At*RibF1 as well as its *C-*terminal domain were submitted to the BioInfoBank Meta Server [44]. The best aligned template provided by FUGUE [45] and PSI-BLAST [37] were subsequently employed to drive homology modelling with Modeller [46]. Further templates were found with HHpred [39] scans of the pdb70 library. Structural superposition and alignments were performed with the software MAMMOTH [47]. Molecular structures and models were inspected, analyzed and plotted with PyMol [48]. Secondary structure predictions were made with PSIPRED [40].

### Production of GmFADS and activity measurement

*GmFADS *gene synthesis, and *E. coli *protein over-expression and purification were carried out by GeneScript USA Inc. Conversion of FMN into RF was qualitatively assayed by addition of *Gm*FADS or *Ca*FADS [11] (final enzyme concentration ~ 0.2 μM) to a solution (final volume, 150 μl) containing 50 μM FMN, either 0 or 0.2 mM ATP and 10 mM MgCl_2_, in 50 mM Tris-HCl, pH 8.0. After incubation overnight at 25°C or 5 min at 37°C the reaction was stopped by boiling the preparations for 5 minutes. Transformation of FMN was visualized by resolving the products of the reaction at room temperature and in the dark by TLC on Silica Gel SIL-G-25 (20 cm × 20 cm, thickness 0.25 mm) plates. The moving phase was a solution of butanol:acetic acid:water (12:3:5). Flavin TLC spots were visually examined and scanned by determining their fluorescence under an ultraviolet light [11].

## List of Abbreviations

FADS-type I: bifunctional prokaryotic enzyme with riboflavin kinase and FMN adenylyltransferase activities; FADS-type II: prokaryotic enzyme with FMN adenylyltransferase activity of FADS in the *N*-terminal module and a putative different activity in the *C*-terminal module; FMNAT: monofunctional prokaryotic enzyme with FMN adenylyltransferase activity; FMNAT-module: module of FADS with FMN adenylyltransferase activity; plant-like FADS: bifunctional enzyme found in plants with FMN adenylyltransferase activity of FADS in the *N*-terminal domain and a putative different activity to that of FADS-type I in the *C*-terminal domain; RFK: monofunctional prokaryotic enzyme with riboflavin kinase activity; RFK-module: module of FADS with riboflavin kinase activity; Tris: Tris (hydroxymethyl)aminomethane.

## Authors' contributions

IY carried out the sequence analysis, sequence alignment and molecular modelling, participated in the design and coordination of the study, and drafted the manuscript. BCM participated in the design of the study, performed the phylogenetic analysis, and helped write the manuscript. SAL participated in the preliminary experimental studies. MM conceived the study, and participated in its design and helped draft the manuscript. All authors have read and approved the final manuscript.

## Supplementary Material

Additional file 1 **Additional Figures.** Figure S1.- Taxonomy of plant-like FADS sequences used in the phylogenetic analysis presented in the main text (Figure 3). Figure S2.- Multiple alignment of a representative set of FADS-type I, FADS-type II and plant-like FADS protein sequences. The alignment was obtained, as explained in Methods, to drive the phylogenetic analysis presented in the paper. Figure S3.- Trimmed multiple alignment of the set of FADS-type I, FADS-type II and plant-like FADS protein sequences used to build the PHYML maximum likelihood tree in Figure 3 of the paper. The alignment was trimmed with the 'automated1' option of the trimAl software. Figure S4.- HHPred alignment of structural template *Sy*NadMNudix (pdb 2qjo) and *At*RibF1. Click here for file

## References

[B1] Müller F Müller F Chemistry and Biochemistry of Flavoenzymes 1991 1 CRC PressBoca Raton, FL 1 77

[B2] Powers HJ Riboflavin (vitamin B-2) and health Am J Clin Nutr 2003 77 1352 1360 12791609 10.1093/ajcn/77.6.1352

[B3] Joosten V van Berkel WJ Flavoenzymes Curr Opin Chem Biol 2007 11 195 202 10.1016/j.cbpa.2007.01.010 17275397

[B4] Van Berkel WJH Chemistry of flavoenzymes Wiley Encyclopedia of Chemical Biology 2008 John Wiley & Sons, Inc, Hoboken, NJ

[B5] Merrill AH Jr Lambeth JD Edmondson DE McCormick DB Formation and mode of action of flavoproteins Annu Rev Nutr 1981 1 281 317 10.1146/annurev.nu.01.070181.001433 6764718

[B6] Bacher A Eberhardt S Fischer M Kis K Richter G Biosynthesis of vitamin B2 (Riboflavin) Annu Rev Nutr 2000 20 153 167 10.1146/annurev.nutr.20.1.153 10940330

[B7] Roje S Vitamin B biosynthesis in plants Phytochemistry 2007 68 1904 1921 10.1016/j.phytochem.2007.03.038 17512961

[B8] Krupa A Sandhya K Srinivasan N Jonnalagadda S A conserved domain in prokaryotic bifunctional FAD synthetases can potentially catalyze nucleotide transfer Trends Biochem Sci 2003 28 9 12 10.1016/S0968-0004(02)00009-9 12517446

[B9] Wang W Kim R Jancarik J Yokota H Kim S-H Crystal structure of a flavin-binding protein from *Thermotoga maritima* Protein Struct Funct Genet 2003 52 633 635 10.1002/prot.10353 12910462

[B10] Wang W Kim R Yokota H Kim S-H Crystal structure of a flavin-binding to FAD synthetase of *Thermotoga maritima* Protein Struct Funct Genet 2005 58 246 248 10.1002/prot.20207 15468322

[B11] Frago S Martínez-Júlvez M Serrano A Medina M Structural analysis of FAD synthetase from *Corynebacterium ammoniagenes* BMC Microbiology 2008 8 160 175 10.1186/1471-2180-8-160 18811972 PMC2573891

[B12] Herguedas B Martínez-Júlvez M Frago S Medina M Hermoso JA Crystallization and preliminary X-ray diffraction studies of FAD synthetase from *Corynebacterium ammoniagenes* Acta Cryst Section F - Structural Biology and Crystallization Communications 2009 65 1285 1288 10.1107/S1744309109044789 PMC2802882 20054130

[B13] Bauer S Kemter K Bacher A Huber R Fischer M Steinbacher S Crystal structure of *Schizosaccharomyces pombe *riboflavin kinase reveals a novel ATP and riboflavin-binding fold J Mol Biol 2003 326 1463 1473 10.1016/S0022-2836(03)00059-7 12595258

[B14] Karthikeyan S Zhou Q Mseeh F Grishin NV Osterman AL Zhang H Crystal structure of human riboflavin kinase reveals a β barrel fold and a novel active site arch Structure 2003 11 265 273 10.1016/S0969-2126(03)00024-8 12623014

[B15] Solovieva IM Kreneva RA Leak DJ Perumov DA The ribR gene encodes a monofunctional riboflavin kinase which is involved in regulation of the *Bacillus subtilis *riboflavin operon Microbiology-SGM 1999 145 67 73 10.1099/13500872-145-1-67 10206712

[B16] Clarebout G Villers C Leclercq R Macrolide resistance gene mreA of *Streptococcus agalactiae *encodes a flavokinase Antimicrob Agents Chemother 2001 45 2280 2286 10.1128/AAC.45.8.2280-2286.2001 11451686 PMC90643

[B17] Kashchenko VE Shavlovskii GM Purification and properties of riboflavin kinase of pichia-guilliermondii Biochemistry-Moscow 1976 41 313 319 6079

[B18] Santos MA Jiménez A Revuelta JL Molecular characterization of FMN1, the structural gene for the monofunctional flavokinase of *Saccharomyces cerevisiae* J Biol Chem 2000 275 28618 28624 10.1074/jbc.M004621200 10887197

[B19] Wu M Repetto B Glerum DM Tzagoloff A Cloning and characterization of FAD1, the structural gene for flavin adenine dinucleotide synthetase of *Saccharomyces cerevisiae* Mol Cell Biol 1995 15 264 271 7799934 10.1128/mcb.15.1.264 PMC231949

[B20] McCormick DB Oka M Bowers-Komro DM Purification and properties of FAD synthetase from liver Methods Enzymol 1997 280 407 413 full_text 9211336 10.1016/s0076-6879(97)80132-2

[B21] Huerta C Borek D Machius M Grishin NV Zhang H Structure and mechanism of an eukaryotic FMN adenylyltransferase J Mol Biol 2009 389 388 400 10.1016/j.jmb.2009.04.022 19375431 PMC2928223

[B22] Brizio C Gallucio M Wait R Torchetti EM Bafunno V Accardi R Gianazza E Indiveri C Barile M Over-expression in *Escherichia coli *and characterization of two recombinant isoforms of human FAD synthetase Biochem Biophys Res Comm 2006 344 1008 1016 10.1016/j.bbrc.2006.04.003 16643857

[B23] Torchetti EM Brizio C Colella M Galluccio M Giancaspero TA Indiveri C Roberti M Barile M Mitochondrial localization of human FAD synthetase isoform 1 Mitochondrion 2010 10 263 273 10.1016/j.mito.2009.12.149 20060505

[B24] Sobhanaditya J Rao NA Affinity-chromatographic procedure for the purification of the enzyme from mung-bean (*Phaseolus aureus*) seeds and conformational-changes on its interaction with ortho-phosphate Biochem J 1981 197 227 232 6274324 10.1042/bj1970227 PMC1163074

[B25] Sadasivam S Shanmugasundaram ER Studies on flavokinase of *Solanum nigrum *L Enzymologia 1966 31 203 208 6005323

[B26] Giri KV Krishnaswamy PR Rao NA Occurrence of flavokinase activity in plants Nature 1957 179 1134 1135 10.1038/1791134b0 13430811

[B27] Giri KV Rao NA Cama HR Kumar AS Studies on flavinadenine dinucleotide-synthesizing enzyme in plants Biochem J 1960 75 381 386 13828163 10.1042/bj0750381 PMC1204435

[B28] Mitsuda H Tsuge H Tomozawa Y Kawai F Multiplicity of acid phosphatase catalyzing FMN hydrolysis in spinach leaves J Vitaminol (Kyoto) 1970 16 52 57 4317626 10.5925/jnsv1954.16.52

[B29] Sandoval FJ Roje S An FMN hydrolase is fused to riboflavin kinase homolog in plants J Biol Chem 2005 280 38337 38345 10.1074/jbc.M500350200 16183635

[B30] Sandoval FJ Zhang Y Roje S Flavin nucleotide metabolism in plants: Monofunctional enzymes synthesize FAD in plastids J Biol Chem 2008 283 30890 30900 10.1074/jbc.M803416200 18713732 PMC2662166

[B31] Giancaspero TA Locato V de Pinto MC de Gara L Barile M The occurrence of riboflavin kinase and FAD synthetase ensures FAD synthesis in tobacco mitochondria and maintenance of cellular redox status FEBS J 2009 276 219 231 10.1111/j.1742-4658.2008.06775.x 19049514

[B32] Lemieux C Otis C Turmel M Ancestral chloroplast genome in *Mesostigma viride *reveals an early branch of green plant evolution Nature 2000 403 649 652 10.1038/35001059 10688199

[B33] Martin W Rujan T Richly E Hansen A Cornelsen S Lins T Leister D Stoebe B Hasegawa M Penny D Evolutionary analysis of *Arabidopsis*, cyanobacterial and chloroplast genomes reveals plastid phylogeny and thousands of cyanobacterial genes in the nucleus Proc Natl Acad Sci USA 2002 99 12246 12251 10.1073/pnas.182432999 12218172 PMC129430

[B34] Marcotte EM Pellegrini M Ng HL Rice DW Yeates TO Eisenberg D Detecting protein function and protein-protein interactions from genome sequences Science 1999 285 751 753 10.1126/science.285.5428.751 10427000

[B35] Huang N Sorci L Zhang X Brautigan C Li X Raffaelli N Magni G Grishin NV Osterman A Zhang H Bifunctional NMN adenylyltransferase/ADP ribose pyrophosphatase: structure and function in bacterial NAD metabolism Structure 2008 16 196 209 10.1016/j.str.2007.11.017 18275811 PMC2258087

[B36] Ogawa T Yoshimura K Miyake H Ishikawa K Ito D Tanabe N Shigeoka S Molecular characterization of organelle-type Nudix hydrolases in *Arabidopsis* Plant Physiol 2008 148 1412 1424 10.1104/pp.108.128413 18815383 PMC2577243

[B37] Altschul SF Madden TL Schaffer AA Zhang J Zhang Z Miller W Lipman DJ Gapped BLAST and PSI-BLAST: a new generation of protein database search programs Nucl Acids Res 1997 25 3389 3402 10.1093/nar/25.17.3389 9254694 PMC146917

[B38] Thompson JD Higgins DG Gibson TJ CLUSTAL W: improving the sensitivity of progressive multiple sequence alignment through sequence weighting, position-specific gap penalties and weight matrix choice Nucl Acids Res 1994 22 4673 4680 10.1093/nar/22.22.4673 7984417 PMC308517

[B39] Söding J Biegert A Lupas AN The HHpred interactive server for protein homology detection and structure prediction Nucl Acids Res 2005 33 W244 W248 10.1093/nar/gki408 15980461 PMC1160169

[B40] McGuffin LJ Bryson K Jones DT The PSIPred protein structure prediction server Bioinformatics 2000 16 404 405 10.1093/bioinformatics/16.4.404 10869041

[B41] Capella-Gutierrez S Silla-Martínez JM Gabaldón T trimAl: a tool for automated alignment trimming in large-scale phylogenetic analyses Bioinformatics 2009 25 1972 1973 10.1093/bioinformatics/btp348 19505945 PMC2712344

[B42] Guindon S Gascuel O A simple, fast and accurate algorithm to estimate large phylogenies by maximum likelihood Syst Biol 2003 52 696 704 10.1080/10635150390235520 14530136

[B43] Abascal F Zardoya R Posada D ProtTest: Selection of best-fit models of protein evolution Bioinformatics 2005 21 2104 2105 10.1093/bioinformatics/bti263 15647292

[B44] Ginalski K Elofsson A Fischer D Rychlewski L 3D-Jury: a simple approach to improve protein structure predictions Bioinformatics 2003 19 1015 1018 10.1093/bioinformatics/btg124 12761065

[B45] Shi J Blundell TL Mizuguchi K FUGUE: Sequence-structure homology recognition using environment-specific substitution tables and structure-dependent gap penalties J Mol Biol 2001 310 243 257 10.1006/jmbi.2001.4762 11419950

[B46] Sali A Blundell AT Comparative protein modelling by satisfaction of spatial restraints J Mol Biol 1993 234 779 815 10.1006/jmbi.1993.1626 8254673

[B47] Ortíz AR Strauss CE Olmea O MAMMOTH (matching molecular models obtained from theory): an automated method for model comparison Protein Sci 2002 11 2606 2621 10.1110/ps.0215902 12381844 PMC2373724

[B48] DeLano WL The PyMOL User's Manual 2002 DeLano Scientific, Palo Alto, CA, USA

