# Supplementary material for: Evolutionary divergence of chloroplast FAD synthetase proteins
Source: BMC Evol Biol. 2010 Oct 18;10:311. doi: 10.1186/1471-2148-10-311 (PMC2972280; doi:10.1186/1471-2148-10-311)
Supplement: Additional file 1 — Additional Figures. Figure S1.- Taxonomy of plant-like FADS sequences used in the phylogenetic analysis presented in the main text (Figure 3). Figure S2.- Multiple alignment of a representative set of FADS-type I, FADS-type II and plant-like FADS protein sequences. The alignment was obtained, as explained in Methods, to drive the phylogenetic analysis presented in the paper. Figure S3.- Trimmed multiple alignment of the set of FADS-type I, FADS-type II and plant-like FADS protein sequences used to build the PHYML maximum likelihood tree in Figure 3 of the paper. The alignment was trimmed with the 'automated1' option of the trimAl software. Figure S4.- HHPred alignment of structural template SyNadMNudix (pdb 2qjo) and AtRibF1. [file 1471-2148-10-311-S1.PDF]

Additional file 1 for the manuscript:

Evolutionary divergence of FAD synthetase proteins in plant chloroplasts

Total figures: 4

Figure S1.- Taxonomy of plant-like FADS sequences used in the phylogenetic analysis presented in the main text (Figure 3).

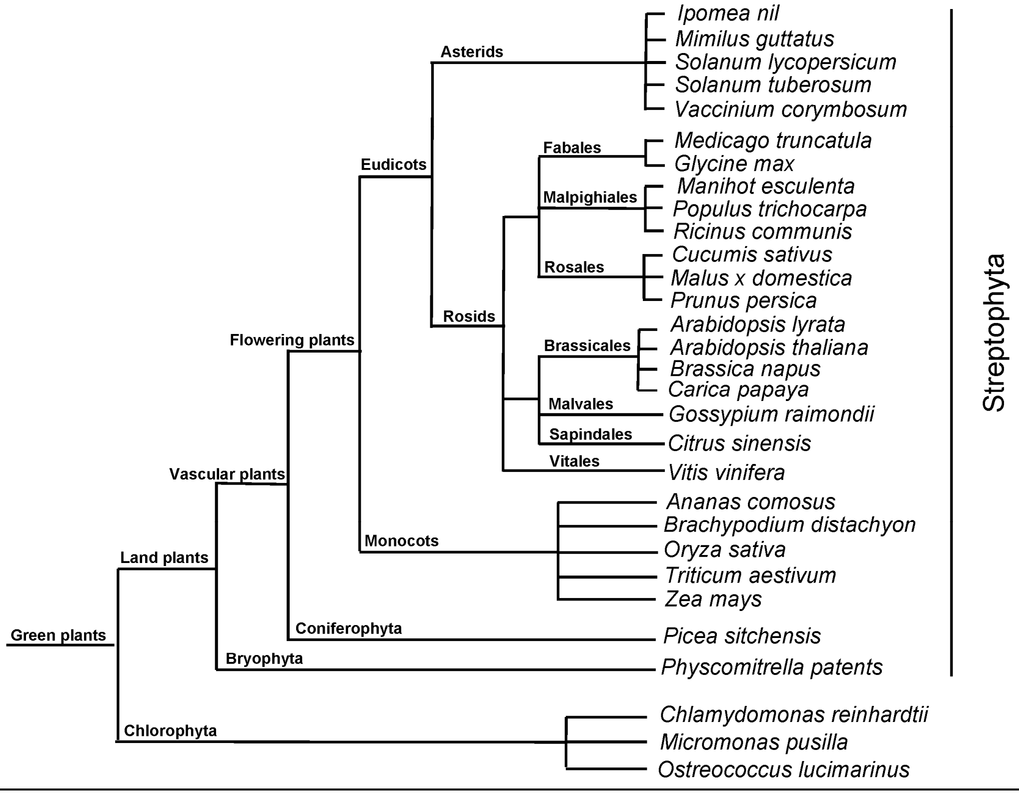

Figure S2.- Multiple alignment of a representative set of FADS-type I, FADS-type II and plant-like FADS protein sequences. This alignment was obtained, as explained in Methods, to drive the phylogenetic analysis presented in the paper.

```
>ss_pred
-----------------------------------------------------CEEEEEC
CCCEEH--HHHHHHHHHHHHHHHC------------------------------------
-----------C--CCEEEEEECCCHHHCCCCCC-----CCCCCHHHHHHHHHHC--CCE
EEEECC--HH---HCCCHHHHHHHHHHC---CE-EEEE----------------------
--------------CCCCCCCCCCCCCHHHHHHH------CCEEEEECCEEEC-------
---------------------------------------------------CEEEEHH-H
HHHHHH-CCCHHHHHHHCCCCEEEEEEEEECCCCC-----------CCCCCCEEECCCCC
CCEEC--CCCCEEEEEEEEC-CCCEEE-E----------EEEC-----CCCCEECCC-CE
E-------EEEEEEEC--CCC------CCCCC--EEEEE-EEEHHCCCCCCCC-HHHHHH
HHHHHHHHH------HHHHHHHHCCCCCCCC--------
>ss_conf
-----------------------------------------------------8888617
775007--8899999999999963------------------------------------
-----------8--95899971798163178888-----5358989999999966--974
998161--76---648988999998656---72-8998----------------------
--------------36664188984797999872------9838985412218-------
---------------------------------------------------8357168-9
999987-4987799986298568998999734115-----------41588443044688
86113--5776479999966-992650-1----------6864-----758703689-41
4-------89999851--573------50775--46663-1103254447899-899999
999989999------9999986367541259--------
>OUTGROUP_AtFHy/RFK_Arabidopsis_thaliana
---------------------------------------------------EFYPLFSAQ
MDKIKSLPGANRLIRHLKCH----------------------------------------
-----------------GVPVALASNSSRANIES----KISYHEGWKECFSVIVG-----
--SDEV-SKG---K-PSPDIFLEAAKRLKKDPA-DCLV----------------------
--------------IEDSVPGVMAGKAAGTKVIAVPSLPKQTHLYTSADEVIN-------
-----------------------------S-----------------------LLDIR-L
EKWGLP-PFQDWIENTLPIDPWHIGGPVIKGFGRG--------S--KVLGIPTANLSTKD
YADELVEHPSGVYFGWAGLA-KR-----------GVFKMVMSI-----GWNPYFNNK-EK
T--------IEPWLLH--DFT-----EDFYGE--ELRLI-IVGYIRPEANFSS-LESLIA
KIHED----------REVAEKALDLPSYAKFKGDPYLTK
>FADS-type_I_Thermotoga_maritima
-----------------------------------------------------MVVSIGV
FDGVHI--GHQKVLRTMKEIAFFR------------------------------------
-----------K--DDSLIYTISYPPEYFLPDFP-----GLLMTVESRVEMLSRY--ART
VVLDFF--RI---KDLTPEGFVERYLSG---VS-AVVV----------------------
--------------GRDFRFGKNASGNASFLRKK------GVEVYEIEDVVVQ-------
---------------------------------------------------GKRVSSS-L
IRNLVQ-EGRVEEIPAYLGRYFEIEGIVHKDREFG-----------RKLGFPTANIDRGN
EKLVD--LKRGVYLVRVHLP-DGKKKF-G----------VMNV-----GFRPTVGDA-RN
V-------KYEVYILD--FEG------DLYGQ--RLKLE-VLKFMRDEKKFDS-IEELKA
AIDQDVKSA------RNMIDDIINSKFEKEG--------
>FADS-type_I_Corynebacterium_ammoniagenes
-----------M------------DIWY-GTAAVPKDLD-N------------SAVTIGV
FDGVHR--GHQKLINATVEKAREV------------------------------------
-----------G--AKAIMVTFDPHPVSVFLPRR--APLGITTLAERFALAESFG--IDG
VLVIDFTREL---SGTSPEKYVEFLLEDTLHAS-HVVV----------------------
--------------GANFTFGENAAGTADSLRQICQSR---LTVDVIDLLDD--------
--------------------------------------------------EGVR-ISSTT
VREFLS-EGDVARANWALGRHFYVTGPVVRGAGRG--------G--KELGFPTANQYFHD
TVAL---PADGVYAGWLTIL-PTEAPVSGNMEPEVAYAAAISV-----GTNPTFGDE-QR
S--------VESFVLD--R------DADLYGH--DVKVE-FVDHVRAMEKFDS-VEQLLE
VMAKDVQKT------RTLLAQDVQAHKMAPETYFLQAES
```

```
>FADS_type_I_Paulinella_chromatophora
-------------------------MLI-PLRSPQEAVK-P------------TAVAVGS
FDGLHK--GHRRVITNISENSTTI------------------------------------
-----------G---VPTVVSFWPHPREVLYGDP---RLRLDMPAEKLTLLESLG--IKQ
LVLVPFSERL---AELTPEIFVRQVLKQQLGAL-KVAV----------------------
--------------GKNFRFGVNRSGDTSALGRIAQEM--GIKVEILPILWD--------
---------------------------------------GN-E----------RVSSS-R
IRRALG-EGKIQEATRLLGRPYRFSGRVVNSSDVK-----------RNHGLPTLKVIVDG
RKFL---PRQGIYAVWVRLD-KG----------DVGLSSGGPI-----GAIMSLGPQ-SL
VDPTIPSQ-VEVYFLD--GRA------DFDEV--RVYIE-PVSLLRGQQEFWG-TEGFYQ
QIHNDIMQA------RKRLEIPNDYNIYD----------
>FADS-type_I_Micrococcus_luteus_NCTC_2665
-----------M------------RVWN-SLDEVPTDLP-R------------TVVTLGN
FDGVHR--GHREVLRRVVELARAR------------------------------------
-----------G--ALAVAVTFTPHPRAVHQPEV--PHVDIISPEQRVVLLEEAG--LDA
VLLQRYTLEF---ADQSPEEFVRGMLVHGLHAA-VVVV----------------------
--------------GHDVRFGRGNTGDVAEMVRLGAHY--GFEVEAVEEFPAE-------
--------------------------------------HGA-E--------PERRCSSTW
VREALD-AGDVAQAAAVLGRHHVLTGEVVHGFARG-----------RELGFPTANLETDV
QGMI---PADGVYAGWVHDA-H-----------GGVWPAAISI-----GSNPTFEDV-SR
V--------VEAHVID--RHDERVEDFDLYGQ--HIEVE-FVARLRGMVAYEG-VEKLVA
QITQDVDEA------RAILATTPSDR-------------
>FADS-type_I_Desulfomicrobium_baculatum_DSM_4028
-----------M------------HCVT-WPDQISGLEK-GS------------CVTIGN
FDGVHI--GHQRLIARVRDLAAGF------------------------------------
-----------G--LPSVVITFEPHPLRFFTGKK--TPPFITLYEQRAELIRSLG--IDH
LLCLEFNQAL---ASMSPEDFVRRILVEGLHIK-ELVI----------------------
--------------GYDYAFGKGRRGNYALLSQLGKQW--AFG---VEQLEPV-------
---------------------------------------MV-D-------QAIVSSTR--
IRDLVE-AGDVWAAKPLLGRFYRVTGTVVHGQNRG--------G--RLLGFPTANVHLV-
DELF---PKTGVYCCWAEL------------DG-EIHQAVANI-----GYNPTFGN--DV
L-------SVEVHVMD--FSA------DLYER--TLKVH-FVQRLRGERKFSG-LDELKA
QIGKDVALA------RTILA-------------------
>FADS-type_I_Mycobacterium_marinum_M
-----------M------------QRWR-GQDEIPTDWG-R------------CVLTVGV
FDGVHR--GHAELIAHAVKAGRAR------------------------------------
-----------G--VPTVMMTFDPHPMEVVYPGS--HPAQLTTLTRRAELVEELG--IDV
FLVMPFTTDF---MKLTPDRFIHELLVEHLHVV-EVVV----------------------
--------------GENFTFGKKAAGSVDTLRHAGERF--GFAVEAMSLVSEH-------
--------------------------------------HSN-E---------TVTFSSTY
IRSCVD-AGDVMAATEALGRPHRVEGVVVRGYGRG-----------AELGFPTANVAPPM
YSAI---PADGVYAAWFTVL-G-HGPVTGAVIPGERYQAAVSV-----GTNPTFSGR-TR
T--------VEAFVLD--TAA------DLYGQ--HVALD-FVARIRGQKKFAS-VPELVA
EIGADTERT------RVLLST------------------
>FADS-type_I_Chlorobium_chlorochromatii_CaD3
-MRLITYDNHHV------------YAYG-SSEPLTFLPQ-P------------SVVTVGS
YDGVHC--GHRVILSRLVEVAHHN------------------------------------
-----------N--LRSVVVTFEPHPRTVLKGALTGPLGLLTTLEEKSDLLAAAA--VDL
LFVVRFTHDF---AARTSDDFIRNVLVGLLGAE-RIIV----------------------
--------------GYDHAFGRDRSGSHNTLERLGNEL--HFGVEVIDEVLI--------
---------------------------------------GN-EH---------LSSTR--
IRKLLQ-DGRIEEVNEFLGSPYLITGWVVQGAQLG-----------RTIGFPTVNLQFHP
AKLL---PRYGVYFARTMVQ-G------------VPYMALMNI-----GKRPTVSSN-GE
A-------TIEAHLLG--FEG------SLYGE--ELRFS-ILRFIRDEKRFAS-LEALQE
QLEKDKKAV------EMYLE-------------------
>FADS-type_I_Chlamydia_trachomatis_D_UW-3_CX
-----------M------------MQMD-LFYSLLPSSN-PV-----------ESVTIGF
FDGCHL--GHQALLSFLTKFPSK-------------------------------------
----------------SGVITFSQHPEHTLSNSP---PETITSLEERVQLLAGCG--IDY
LAVLPFNQEI---ANQEAEPFIQSIYKT-LRPS-RIVL----------------------
--------------GYDSRLGKGGLGTAQTLRPFAASL--GISLEEVPPLQIE-------
```

```
-----------------------------GT---------------------IVSSRK--
IRQFLR-KKDLCSAEKFLGRPFSYTGKVAHGRGIG-----------ASFGYATINLP-LT
HSLL---PL-GVYTCTIV-------------IEGFSYAGVMNL-----GMAPTMQR--HQ
L-------CLEAHILD--FSE------DLYDK--SITVI-PEQFLREEKLFSS-KDELVL
AIQEDIRQA------RLNKNR------------------
>FADS-type_I_Bacillus_subtilis_subsp_subtilis_str_168
-----------M------------KTIH-ITHPHHLIKE-EQAK---------SVMALGY
FDGVHL--GHQKVIGTAKQIAEEK------------------------------------
-----------G--LTLAVMTFHPHPSHVLGRDK-EPKDLITPLEDKINQIEQLG--TEV
LYVVKFNEVF---ASLSPKQFIDQYIIG-LNVQ-HAVA----------------------
--------------GFDFTYGKYGKGTMKTMPDDLDGK---AGCTMVE-KLTE-------
-----------------------------QDK--------------------KISSSY--
IRTALQ-NGDVELANVLLGQPYFIKGIVIHGDKRG-----------RTIGFPTANVGLNN
SYIVP--PT-GVYAVKAEVN-------------GEVYNGVCNI-----GYKPTFYEK-RP
E-----QPSIEVNLFD--FNQ------EVYGA--AIKIE-WYKRIRSERKFNG-IKELTE
QIEKDKQEA------IRYFSNLRK---------------
>FADS-type_I_Clostridium_thermocellum_ATCC_27405
------------------------MRVI-YGAESNYNFT-RS-----------TGVGLGN
FDGLHV--GHMALIDKLIKESESN------------------------------------
-----------S--LDSVVYTFSKHPENIIRKEL--FTPLITSTRKKVELLEKTR--LDY
LYFEKFDETF---SRMRPEDFVKDILVERLNMK-LAVA----------------------
--------------GFNYRFGYRGMGDTELLRKLGRKY--GFRVIVIEPVML--------
---------------------------------------GD-E---------VVSSTK--
IRNYIL-EGDMERVFAFLGRHYSVAGKVEKGRRVG-----------NTIGFPTANIYPED
YLVL---PCHGVYITRT----L---------VDGKMYHSITNV-----GNNPTFGGV-DR
I-------SVETYIFD--FEK------DIYGK--EIEVF-FISKIREEMRFGS-VEELIE
QIHKDIEIA------KKVLAEDL----------------
>FADS-type_I_Lactobacillus_fermentum_IFO_3956
-----------M------------EVIK-VHHPLLEQQV-PDGP---------VVVAMGF
FDGVHR--GHQAVIARAKEEAVRR------------------------------------
-----------Q--VPLAVLTYDKLPGIVYQRYD-EGVHYLTTIDRKLALLEQLG--ADL
VYLVDFTAKL---GSLSPEEFVSDYLVR-MHAV-AVVA----------------------
--------------GFDHTYGKKDVATMDRLVGYAAGR---FDVVTVK-KQET-------
-----------------------------RGA--------------------KIGSSR--
IRDLID-QGRVEDANQLLGYRYQTSGIVVHGLARG-----------RTIGFPTANVAWDP
LERI---PAVGVYTVNFLVD-------------GHWYGGMASV-----GYNVTFGQN---
K-----HKTIEVYLFD--FKG------NIYGE--HVTVS-WIQRLRGEIKFDG-VDGLID
QLHADQINS------LAILRNPPTLPAGPLN--------
>FADS-type_I_Mycoplasma_penetrans_HF-2
--------------------------------MKIIKIN-LP-----------FNNQIEN
TENLVL--GHFNLIHYGHHELFKE------------------------------------
-----------L--KNFSFLIFENNPSKFKR---------PYSLDERIENLSKFN--PEY
IFVYDILKNN---I--DADVFIKEVLLK-IKPK-NIVV----------------------
--------------GSDFCFGKNKKGNVELLKS-------FFNLKEIYKNEFY-------
-------------------------------------------------------SSRN-
IIELIE-SGFLEKANEMMMFNFYYSNVVIKGKGLA-----------SELDVPTANIEDNK
DIKI---PS-GSYSSITL-------------IDDKLYKSISFI-----GIPKSFENT-KP
--------TVETHIFD--FNQ------DIYNK--KIKIY-PIKFIRPNQKFDD-IKTLIK
FIKNDCDIA------KKFLSDFDLKKIKY----------
>FADS-type_I_Chloroflexus_aggregans_DSM_9485
-----------M------------QIAR---DLVPGLAN-RA-----------TVLTIGR
FDGVHL--GHQQLIRTTVERARAL------------------------------------
-----------D--MLSAVLTWEPNPRAVLQPGQ--PLQLLTDLDEKIEQIRRLE--PDL
LIIAPFTNEI---RQLSAAEYMARICAA-VPVR-EIWV----------------------
--------------GEDFAMGRGREGDIPRLMEIGREL--GFA---VGALSKY-------
-----------------------------MMA------------------GIPVSSSR--
IRELVL-AGNVAGAGALLGRPFALRGLVTRGDGRG-----------RQIGFPTANLQVNP
DVVV---PAHGVYACRTYL------------ATGEVVPTVTNI-----GVRPTFDG--TR
Q-------VVEAHLID--WQG------DLYDQ--HLRIE-LLMRLRNERKFSG-IDELVA
QIRHDVAEA------RVVLGVPTTS--------------
```

```
>FADS-type_I_Rhodospirillum_centenum_SW
-----------M------------KLYR-HTTDLAPELR-G------------CAVALGN
FDGVHR--GHRAVIAATQEKAAAL------------------------------------
-----------G--GATVVLTFEPHPRSLFRPGD--PPFRLTPFRIKARLIEALG--VDA
LFVIHFDEAF---SRRTAEEFVEHVLIQELGAA-HVVA----------------------
--------------GYDFVFGHKRGGDMALLRRMGEER--GFGVTEVRPVADA-------
-----------------------------GGT--------------------VFSSTR--
VRELLQ-AGEPQAASGILGHPFELEGRVEHGDKRG-----------RTIGFPTANVELG-
EYLR---PRFGVYAVLAGID-Q--------GGGTVWHKGVANL-----GRRPTVGGH---
------VERLEVHLFD--FDG------DLYGR--HLRVQ-LLHFLRPELKFDG-LDALKA
QIAADALAA------RGLLAAEG----------------
>FADS-type_I_Desulfobacterium_autotrophicum_HRM2
-----------M------------ELIE-DLKFIKTPFN-KA------------VITIGN
FDGVHK--GHQAIFHQVIEKAEEI------------------------------------
-----------G--GTSVVMTFDPHPLKVL-GHN--GPPLITRKDQKIELISATG--IDK
ILCLPFTREF---AAISAQEFIKDLLINQLGMK-AIVV----------------------
--------------GLDYSFGRNREGNLELMQKAGKQL--GFEVLIADWINDT-------
-----------------------------ETG------------------SERISSTR--
IRELVM-EGRVDETPKFLGRFYQIRGKVVKGRQRG--------G--SKLGFPTANIKLH-
DELS---PKMGVYAVTVET------------QT-GKFKGVANI-----GYSPTFDD--HI
F-------TIEVHLLD--FAG------DLYNT--RIRIN-MIKRLRDEKKFSN-LEELSN
QIRNDIQLA------REILA-------------------
>FADS-type_II_Alistipes_putredinis_DSM_17216
-----------M------------RVFH-GFENLPSFRS-P-------------AATVGS
YDGVHS--GHRVLLDRIRREAAAVG-----------------------------------
--------------GESIVLTFAPHPRVTLGTQER--LRLLTSLEEKIYLLDRFG--IDN
LIVIPFDRAF---SRIPSESFVKDYLIGKVGVK-NLVV----------------------
--------------GFNHRFGHDKEGDYRLLNGLHDEF--GFRVTEIEKQDVD-------
-----------------------------AEK---------------------VSSTV--
IRRLIE-RGEMNKAARMLSHPYLLAGDVD---------------------CAGHIASGEA
LKLL---PPPGEYPVRIEGR-P----------------GVLRITAK--G-----------
--------TPELLRTA--GKM-------------PSGHI-LIEF----------------
---------------------------------------
>FADS-type_II_Eubacterium_saphenum_ATCC_49989
---------------------------M-YKGLENIK-K-RS-----------RAVALGN
FDGVHI--AHVKLIDMIKKRARAF------------------------------------
-----------N--LEACVFTFKNHPKEFIPGSE--ILKYINDTEKTMEIFENLG--IDS
LVMVPFDEVL---QRMSPEDFVKDVIVERLGAK-YVCV----------------------
--------------GYDYRFGFEGKGDVSLLDRLGKTY--GFHVDVIDKVSVG-------
-----------------------------GV---------------------TVSSSR--
IRKLIY-EGDFEGVQKLLGRRYMIAGKVVHGKSIG-----------RKLGFRTLNIMPRE
NLCI---PSDGVYYVTRTRV--A--------MKSKVYDSITNI-----GTAPTFKG----
-DAV----SIETNVFD--FNE------DIYGD--IVHID-FLKKIRDEIKFDT-PEELSK
QIAEDVRLA------KDIHSEE-----------------
>FADS-type_II_Arthrobacter_chlorophenolicus_A6
-----------M------------KIWN-SLEDVPAETG-Q------------TVVSLGC
FDGVHR--GHQLLLARLLNAAETN------------------------------------
-----------A--AKSVVVTFDPLPAQLLYPET--APLAVMALEDRLAALEVEG--VDA
VLVIRYTRDV---AAQAAEVFVEKVFVKTLHAA-AVVV----------------------
--------------GEDCRFGQGRAGNIETLRAAGLRW--SFDVSVLGDRTAG-------
---------------------------------------GT-E---------GRRYSSTW
VREVLS-SGDVGIAEQILGRPHRIRMEVTRAAGSW-----------RTK-------ARAV
QGML---PASGDYAGWARVG-S-----------GERIPAIIRI---PRRASLAT------
-DI-----TVELQMPA--DR----YAEKS-PG--MSRKI-SFDFSSTS------------
---------------------------------------
>FADS-type_I_Arthrobacter_chlorophenolicus_A6
----------MV------------YIWH-DPSEVPADFG-P------------SVVTFGN
FDGVHR--GHQQVLSQLIRSARLT------------------------------------
-----------H--SKAVAVTFDPHPAVIHRPES--APRLIMGLEDKLEALGELG--LDA
ILVVKYSLDL---ASLTAEEFVEQYLVDCLHAS-HVVI----------------------
--------------GHDARFGRGNSGDLETMKALGGKF--GFEVQVISEFGSE-------
```

```
-----------------------------GYPL--HDDDGT-D----------RRCSSTW
VREALQ-EGDVATAAEVLGRAHRMRGEVVHGAARG-----------RALGFPTANLASDS
TGLI---PADGIYAGWLVDE-A-----------GKRWPAAISV-----GSNPTF------
-DGV--SRQVEAHVID--RPRE--AVEDF--------DL-YGQTVIVEFVARLRGMVAYR
G-PEALVDQMKLDVVQA------HQLLARH---------
>FADS-type_II_Lactobacillus_plantarum_JDM1
-----------M------------QVIN-LDAHRLKMLA-SQPP---------LVLALGF
FDGVHQ--GHQRVIQTAKRIAKQR------------------------------------
-------------DLPLAVMTFNRHASQLFKSQT--TFRYLNTVAQKSQHMAALQ--VDR
LYITDFNHQF---AGLTPTAFIKDYLVG-LNAQ-VVVA----------------------
--------------GFDYTFGQGGANGMRELAELGAPY---FETVTVDRLANQ-------
-----------------------------QLK---------------------VSSTR--
IRGLIA-RGQIEQANDLLGYTYATQATFDPLT--------------RTIQLANRQQQL--
-------PAAGDYRCWLVSA-------------NYRQAVILRV-----TTTLKIISP---
Y-----QLPPETSVLD----V------DVQWQ--QRALQ-VVSSVSAQHQQS--------
QYSKA----------------------------------
>FADS-type_I_Lactobacillus_plantarum_JDM1
-----------M------------QVIN-LKYPVALSQI-PEGP---------IVLALGF
FDGVHR--GHQQVVATARQAAQAQ------------------------------------
-----------H--AKLAVMTFDQHPSVVFKHTDPQQVRYLTTIDQKTALMSELG--VDI
LYVLHFDATV---GAMPPQTFVDQLIVG-LHAQ-TVVA----------------------
--------------GFDYTYGPAEIANMQRLNDYGHNR---FEIIEVP-KAEL-------
-----------------------------DAE--------------------KISSTR--
IRRALD-AGDIDTANRLLGYQYETAGEVVHGEARG-----------RTLGFPTANVAHGP
NTRV---PGIGIYATMVQIG-------------TRWVMGMASV-----GRNVTFGDH---
-----RPITVEIYLLD--FRG------DLYGH--NLTVR-WGHRMRGEIKFAG-ADALVA
QLKRDEQNTRDYYVAHPFVVTPRIQVAQQSNRLEAQ---
>FADS-type_II_Listeria_monocytogenes_HCC23
-----------M------------EVSH-VTLAPNKDSR-P------------AVLTIGK
FDGVHI--GHQTILNTALSIKKEN------------------------------------
--------------EILTAISFSPHPLWALKQIE-IYREMLTPRMEKERWLAHYG--VDH
LIETAFTPRY---AETTPEEFVRDHLTN-LNLS-HIVV----------------------
--------------GSEFNFGKGRDSDVDLLRDLCKPY--DIGVTSVPVIETN-------
-----------------------------QTK---------------------ISSTN--
IRAFIR-RGHFQEAEQLLGHPWYITGNVENGEMIG-----------------------LD
DYVL---PATGTYQTDS---------------------GMVNVTN---------------
----QRTILVELSDGL--QQLHMKNELS--------------------------------
---------------------------------------
>FADS-type_I_Listeria_monocytogenes_HCC23
-----------M------------KTIY-LHHPITTDEW-TSIK---------KVMALGF
FDGVHL--GHQAVIKKAKQIAEQK------------------------------------
-----------G--LQTAVLTFDPHPSVVLSNIR-KQVKYLTPLEDKAEKMAELG--VDI
MYVVRFTTQF---SELSPQSFVDNYLVA-LNVE-HVVA----------------------
--------------GFDYSYGKKGEGKMTDLAQYADGR---FEVTIVD-KQTA-------
-----------------------------ASD--------------------KISSTN--
IRRAIT-EGELEEANQLLGYPYTTKGTVIHGDKRG-----------RTIGFPTANIRVNE
DYLI---PKLGVYAVKFRVN-------------GETHLGMASI-----GYNITFK-----
-DD-Q-ALSIEVYILD--FHREIYGEEAEIEW--YQFFR-PELKFNG-VEGLIAQLEKDE
QDT------------RAYFADLED---------------
>FADS-type_II_Treponema_denticola_ATCC_35405
--------MDDF------------KIIS-WEGLVEAAQS-VLTGSDFFPEGKETAISVGG
FDGPHK--GHDKLLRQVLTYA----A----------------------------------
-----------ENTLVPGLVTFFRSPAAVKNKAY---SGDVSSLRLRLKKFQELG--FHF
IVLIDFSASF---AKIEGTAFFD-ILIKTIRMK-YLAV----------------------
--------------GSDFFCGYRRGLGVDDLKKIAPQK--GFCFDSIDPVNRG-------
-----------------------------SLK---------------------ISSSA--
IREAVR-LGDFSLAKELLGYPFLFDFVSLPWEVK------------------------DK
NSIFA--PK--AYISQILPQ-S-----------GKYRVLVQKT-----------------
-------DRQEQE-------AHCLINDD--GL--LLCFP-----------------EKD-
SKAFDLKD-LNNF-DTIEFICKE----------------
```

```
>FADS-type_I_Treponema_denticola_ATCC_35405
-----------M------------RITD-KLQPITNGE---------------SCVAVGF
FDGLHV--GHRAVIDRLCKCKNLR------------------------------------
----------------PVLISLSNNSSPVIY-----------TEEEKSYLLQ-NGK-LDT
MFSLS-EDII---KNMTAESFAHDVLNKMLNTK-TLVA----------------------
--------------GENALFG-LDQVDVNHFRSIGKKY--GFTVETVPMECIN-------
---------------------------------------GL-E----------VSSNT--
IKQTIH-DGDFSKVSSMLGSSYLISGTVVHGKGAG-----------HKFGMPTANISIAA
NKLF---PPHGVYGSISRFQ-------------GENHFGMTNI-----GLRPSDDDI-PI
P-------TIETFLLN--FDR------DIYGQ--KVFLE-LLVYIRGIRKFDGGLAEVRQ
QIDKDIKQIHSYIEENGNLLQVKPKEIC-----------
>FADS-type_II_Treponema_pallidum_subsp_pallidum_str_Nichols
-----------M------------RIFR-WSQLQEGACI-ACD--------RGAAISVGG
FDGPHR--GHAFLFDKVFAAACAPVA----------------------------------
-----------DRARCTGLITFT-HPPRKHKTSS--YEGDLSTLRLRLRYFRARG--FDF
VVLIDFSKDF---ARIPGGVFFN-TLLRAVRVC-YLAV----------------------
--------------GVDFRCGHGLDTGVRELRRLGDAH--SFVCDAVGHYTLE-------
---------------------------------------GV-R----------VSSSA--
VRRAVR-CADFESARRLLGRAFSLDGEVIPWQQSG--------GCARTLCAERGRVSQTL
-------PPEGEYAVRLVQG-S--------------------------GVGLRAQLS-VG
SSLVQLRLGTEALSHG----REALDSIEFE------------------------------
---------------------------------------
>FADS-type_I_Synechococcus_sp_JA-3-3Ab_CYA_2407
-----------M------------HVTS-DLAQTLRP----------------TAVAIGN
FDGLHL--GHQKVLQPIRDS--------------LQGVRT--------------------
------------------VLTFHPHPQEVLTGRR---QLLLTPPAEKLALLAQMG--FEQ
VVLLPFTPAF---ARQPPQEFIQTVLEQGLRVR-HLSV----------------------
--------------GWDFCFGHRRSGNAQTLQAWGSER--CIPVEVIPEAQ---------
--------------------------------------LKG-E---------RVSSSR--
IRAALA-TGEVAAATELLGRPYRLIGEVVPGDRRG-----------RELGFPTANLRLPP
EKFLP---RDGVYSVWVNVP-AEAAALP----------GVMNI-----GHRPTFA-G-LQ
HT-------VEVHLLD--WTG------DLYGQ--EVQVA-LQGFIRPERRFPS-VADLIQ
QIRQDCQTA------RQQLGLAEQVGLV-----------
>FADS-type_I_Gloeobacter_violaceus_PCC_7421
-----------M------------AVFR-SLSEVATP----------------CAVALGN
FDGVHL--GHQAVIQAVLG---------------RAGIPT--------------------
------------------VLTFDPHPREYFTGKTG---FLLAPERERTAAILALG--IAQ
VLVLPFDELL---AATEAGAFVEQVLVAGLGAR-FVSV----------------------
--------------GWNFRFGKERAGTTEMLQSYARAG--AFDIEILAERQ---------
--------------------------------------VRG-R---------RVSSSV--
IREALG-CGDLDLARLLLGRAYGLEGEVVRGDQRG-----------RLLGFPTANLQVSG
RKFLP---KDGVYLVSARWG-VQQRWG------------LLNL-----GLRPTFD-G-LK
RT-------IEVHVLD--WEG------DLYGQ--HIKIT-LERYLRPEQKFGS-PGELVA
QLHRDREAAL-----KEIAGNP-----------------
>FADS-type_I_Thermosynechococcus_elongatus_BP-1
------MSKCFL------------CQFK-SGVPLQTP----------------TAIALGN
FDGVHR--GHQEVIRTLLKAAP------------PDCYPS--------------------
------------------VMTFSPHPQAFFTGEE---RLLLTPEAEKRALLHQCG--IAQ
VIVLPFTQAL---AQLSPLEFVEQILVQQLQAK-VLSV----------------------
--------------GFNFGFGRGRSGTAEDLRSLCAPF--GIVVHIVPPYC---------
--------------------------------------RGS-D---------RVSSSA--
VRAALA-AGEVALARELLGRAYTLTGTVTQGEQLG-----------RQLGFPTANLVLPR
EKLL---PRYGVYACRVSGP-ALPREH----------LGVVNI-----GVRPTVG-G-QE
VR-------TEVHLLH--WQG------NLYNQ--EITLH-LEAFIRPELTLPS-LAALRA
QIAADCQVA------VDLLERVVSYA-------------
>FADS-type_I_Acaryochloris_marina_MBIC11017
-----------M------------WVTS-SLETVRTP----------------TVIALGN
FDGIHQ--GHCQVIQSLLSNR-------------SDTAQV--------------------
-----------------TVVAFNPHPQAFFSGEH---QPLLTPLSEKATLLENLG--VDQ
LVLIPFDQAL---ANLSPQDFVKDILVAQLQAQ-FISV----------------------
--------------GFNFCFGYQRAGTAEDLVAIAGQY--QIPVS-ITAPQ---------
```

```
-------------------------------------TNGS-E---------PISSSA--
IREALL-EGNLEKAQQMLGRAYDLTGTVVKGQQLG-----------RTLGFPTANLQVPT
DKFC---PRTGVYSVSVTSN-LWSQPQ----------PGVMNW-----GCRPTVD-G-QQ
PS-------LEVHLLD--WSG------DLYGH--TVTVH-LQQFLRSEQKFAS-LADLKA
QIQADCEIA------RASLAAVV----------------
>FADS-type_I_Synechocystis_sp_PCC_6803
-----------M------------RILS-TTNVLQTP----------------TAIALGN
FDGVHR--GHGVVLRQVMDFAQAV----------EHLLHP--------------------
-----------------AVVSFNPHPRSFFSGRT---QPLLTPLPEKAAQLTAIG--IEQ
LVLLPFTEKL---ANLSPKQFVQSILVEQLQAK-FISV----------------------
--------------GQDFCFGHQRRGNVQDLQNLGQEF--GITVAIAQLEQ---------
--------------------------------------TDT-E---------RISSSR--
IRRALK-EGKLAMANHLLGRPYALRGTVVQGQQLG-----------RKLGFPTANLCLPA
DKLW---PKYGVYAGWVNLN-A--LDV--------PIPAVINL-----GDRPTVN-G-QE
PS-------AEVHLLQ--WSG------DLYGQ--GLEVA-LTHYLRPETKFAG-LDQLKN
QIAQDCQQA------EKLLNLDG----------------
>FADS-type_I_Trichodesmium_erythraeum_IMS101
-----------M------------WITS-SLATALTP----------------TAVALGN
FDGLHQ--GHRQVIEPILNLKGRSKLLSVCPSELTRTTEVDEDTKLKLWHQDSKLSSWHF
ENELGKNDDYLRQKIYSTVVTFNPHPQEFFSGKP---KKLLAPLEEKLAIFKHIG--VEQ
VVLLPFDRNL---ADLTPNKFVEEILVKGLQVH-QISV----------------------
--------------GCDFCFGQKRAGKAKDLQAIAGYY--DIDVTTVPLYH---------
-------------------------------------SENG-E---------RISSSI--
IRQALE-KGDLDKSNRLLGRPYSLIGEVVHGQQLG-----------RTLGFPTANLQLAA
QKFL---PRFGVYAVEVYLS-RQKQPQEIL-HAESPYLGVMNV-----GCRPTLN-S-LD
PT-------VEVHLLD--MSQ------DLYGQ--TLTVN-LIEFLRPEQKFAS-LDLLKA
QIYEDCRIA------RSILKNQTI---------------
>FADS-type_I_Prochlorococcus_marinus_str_NATL1A
MPSKHKNCTFIL------------IPLC-APENAKLP----------------TALALGS
FDGLHL--GHKKVINAILK--------------EPIGVPT--------------------
------------------VVSFWPHPREVLFGES---RLRLDLPNEKTFLLEPLG--IEQ
LVLVPFNKNL---ASKSAETFVEEVLVKTLHAK-HIAV----------------------
--------------GENFRFGRNREGDTSTLKKIGTSL--GIKISIVPIVE---------
--------------------------------------DNH-G---------RLSSSR--
VRKALN-DGDLKHAKHLLERPYTFSGTVEKGRGLG-----------KKIGWPTANLKIDG
RKFLP---SLGVYAAWASIA-NKKERF----------LAVMNM-----GPQPTIDPN-SL
SA-------VEVHLLD--KEI------NLLGH--ELIIE-PVQRIRLQKKFDN-IESLSK
QISSDAKLA------KEILTQKL----------------
>FADS-type_I_Nostoc_punctiforme_ATCC29133
-----------M------------WVAS-SSEGLLTP----------------TAVALGK
FDGVHL--GHQRVIQPVLHPCDRLS-----VASSPQSKENQQQT----------------
----------NQEYIYSTVVTFDPHPQEFFTGQP---RTLLTPLDEKVQQLRSLG--VEQ
LVLLPFDKEL---SALTPEEFVQKILVQQLQCQ-RISI----------------------
--------------GQDFCFGEKRSGTAKDLQLIAAKH--NIPVTIVPLQTYT-------
-----------------------------GDSPTQSSCVST-NP---T-QDARISTSL--
IRQTLE-QGDIENANLLLGRPYTLFGVVVQGQQLG-----------RTIGFPTANLELPK
EKFL---PRQGVYAVRVFTL-SETSNA-----ASSESLGVMNI-----GNRPTVN-G-TY
SS-------AEVHLFD--WSG------DLYGK--KLAIE-LVKFLRPEQKFPS-LEALKT
QIQLDCVVA------KEVLSAEWEQ--------------
>ss_pred
-----------------------------------------------CCCC---EEEEEC
CCCEEH--HHH-HHHHHHHHHCCCEEEEECCCCHHHCCCC--------------------
------------------------------------CCCCCCCCHHHHHHHHHHCCC-CE
EEEEECCHHHHHHHCCHHHHHHHH-HHHHCCCC-EEEE----------------------
--------------CCCCCCCCCCCCCCHHHHHHHHHC--CCEEEEECCE----------
------------------------------EECCCCCCCCC-CCCCCCCCCCEEEHHH--
HHHHHH-CCCHHHHHHHCCCCEEEEEEEE---------ECCCCCCCCCCCCCHHHHHH--
---CC--CCCCEEEEEEEE--CCE-EEEE----------EEEE-----EECCCEEE--EE
EE--------EECCCC--CCC---------CC--EEEEE-EEECC---------------
---------------------------------------
>ss_conf
```

```
-----------------------------------------------9971---899805
565007--889-9999998628736987527808745899--------------------
------------------------------------888646898999999996799-89
999708969963207898999999-99841933-8998----------------------
--------------377652889857989999999862--9759996311----------
------------------------------10663012222-2222223674040588--
998977-4987799986298569998999---------63425657889997243541--
---68--878548999998--790-3201----------3860-----27897007--99
99--------851565--627---------75--68999-88319---------------
---------------------------------------
>plant-like_FADS_Arabidospsis_thaliana_AtRibF1
-----------------------------------------------VAGG---IVALGK
FDALHI--GHR-ELAIQAARIGTPYLLSFVGLAEVLGWKP--------------------
------------------------------------RAPIVAKCDRKRVLSSWASYC-GN
IAPVEFEIEFASVRHLNPQQFVEK-LSRELRVC-GVVA----------------------
--------------GENYRFGYRASGDASELVRLCKDF--GISAYIINSV----------
------------------------------MDKNQVSVNTE-EEDSKSKERGQVSSTR--
VRHALA-AGDVRYVTELLGRPHRVISRTR---------TQDLTSKRGRISLQTSSLLN--
---LP--PGNGVYKACSLI--VGD-KHPI----------SCKV-----IVDTSNLY--IE
TE--------EERFHN--SDE---------SQ--EFQLL-GIEFG---------------
---------------------------------------
>plant-like_FADS_Ipomoea_nil
----------------------------DSLSRSEDGEEPPQERLPFVAGG---IVALGK
FDALHI--GHR-ELAIQAAKIGVPFLLSFVGMAEVLGWEL--------------------
------------------------------------RVPIVAKCDRKRVLSSWAPYC-GG
MIPREFQIEFSKVRYLTPRDFVEK-LSKDLGVL-GVVA----------------------
--------------GENYRFGYKASGDASDLLRLCNEY--GIQACIINSV----------
------------------------------MDKNQDPS-SLYSY-DAK-ERGQVSSTR--
VRYALA-KGDMKYVSELLGRHHRLFLMVGDQEKFT--------SDRCRVSAPKSCLLN--
---LS--PREGVYENCSIM--IDEK------------VIACRV-----VIDTTHIH--LD
WD---------KHTC----IT---------CQ--QLNLV-GID-----------------
---------------------------------------
>plant-like_FADS_Zea_mays
-----------------------------------------------VLGG---IVALGK
FDALHI--GHR-ELAMHASKAGTPFLLSFVGMAEVLGWTY--------------------
------------------------------------RPPIVAHCDRKRVLSSWAPYC-RN
VVPLEYQVEFSKVRSLSPRQFVER-LSKDLRIK-GVVA----------------------
--------------GENYRFGYKASGDATELVKLCEEF--GLSAFIVRSV----------
------------------------------MDTAKRSLNGFSGAINSS-DKGQVSSSR--
VRHALA-MGDMEYVSELLGRKHRLLLMVN----------QHCLHEKKSIVLPNSCMLN--
---MP--PAEGLYENCDLVN--GGYLGP------------CRV-----IINSDTII--IE
MKD-------DNSLS---LNP---------IQ--E-VQL-GIEFG---------------
---------------------------------------
>plant-like_FADS_Medicago_truncatula
IP----------------------LLFDSSFSQQEDDREIISDGISAVAGG---IVALGK
FDALHI--GHR-ELAIQASRAGPPFLLSFVGMAKVFGWEH--------------------
------------------------------------RAPIVAKCDRKRVLSSWFPYC-GN
MVPEEFEIEFSSVRHLSPRQFVEK-LAKELRVR-GVVA----------------------
--------------GENYRFGYKAAGDASELQKLCEEY--EMEAYIIKSV----------
------------------------------MDKN-QYSTNINSSIGSK-ERGQVSSTR--
VREALA-VGDMRYVSELLGRQHRLVLMATDRETFS------FSQYK--VSAPKSCLLN--
---LA--PKEGLYEKCS-----LLLGQEN--------VMLCRV-----IIDSKFVH--IE
TD--------YGGSSD--IFG---------TH--DLQYL-LIEFGDSST-----------
---------------------------------------
>plant-like_FADS_Vitis_vinifera
-----------------------------------------------TVGG---IVALGK
FDALHI--GHR-ELAIQASRVGAPFLLSFVGMAEVLGWEP--------------------
------------------------------------RAPIVAKCDRKRVLSSWAPLC-GN
VTPLEFLIEFSSVRHLTPRQFVEK-LYKELGVR-GVVA----------------------
--------------GENYRFGYKAAGDSSELVRLCEEF--GMGAYIIKPV----------
------------------------------MDNNQDSRNTA-SSNS-K-ERGQVSSTR--
```

```
VRHALS-IGDMKYVSELLGRQHRLILTAQY--------QEAFTSSKHKVSAPKSCLLN--
---LP--PKEGLYENCSLL---IDDENPV----------SCRV-----VIDTTHIH--LE
FD--------EVGPYK--HLT---------SQ--GPWQL-GVEFGV--------------
---------------------------------------
>plant-like_FADS_Prunus_persica
-------------------------------------------------------VALGK
FDALHI--GHR-ELAIQASKVGPPFLLSFVGMAEVLGWEP--------------------
------------------------------------RAPIVARCDRKRVLSSWAPYC-GN
MAPTEFQIEFSSVRHLTPRQFVEK-LLKELGVR-GVVAGKLIRNPLQTILCVSWYERSIA
LIYIEMDLQWVILAGENYRFGYKAAGDATELVRLCEEY--GIGAYIINSV----------
------------------------------MDKNDY--SININSSDVK-DRGQVSSTR--
VRRALA-VGDMKYVSQLLGRQHRLILMA-KEQEGFR-------CCKNKLSVPKSCLLN--
---LG--PKEGLYEKCYVF--IGEDDE----------LLSCNV-----VIDTEYVH--IE
MD--------EVGAS-CNVVG---------TQ--DLSLLR-IEFG---------------
---------------------------------------
>plant-like_FADS_Carica_papaya
-----------------------------------------------VAGG---IVALGK
FDALHV--GHR-ELAIQASKVGAPYLLSFVGMAEVLGWEP--------------------
------------------------------------RAPVVAKCDRKRVLSSWAPYS--N
IAPAEFEIEFSTVRHLAPRQFVEK-LSKELRVS-GVVA----------------------
--------------GENYRFGYKAAGDSSELVRLCQEY--GIGAYIINSV----------
------------------------------MDKNQDSRDID-SNDL-K-ERGQVSSTR--
VRRALA-VGDMDYVSELLGRPHRLILVVKD--H-----KELTSSGKFRMSVPKSYLLN--
---LP--PREGFYDHCCLL--FGDEK-PL----------KCRV-----LIDTTHIH--LE
MD--------EVICN---SKK---------SQ--DYWFL-GIEFGKLS------------
---------------------------------------
>plant-like_FADS_Cucumis_sativus
------------------------------------------------------------
-------------------MVGSPFLLSFVGIAEVLGWEP--------------------
------------------------------------RAPIVAQCDRQRVLSSWAPYC-QN
SAPSEYRIQFSSVRYLTPREFVEK-LSKELRVC-GVVA----------------------
--------------GESYRFGYKAAGDAAELVKLCEEY--GISAYIIKSV----------
------------------------------MDRNQK----VVNSANSK-ERGQVSSTR--
VRHALS-KGDMKYVSELLGRRHRLILMAEGLEGFS--------NSNNRVSAPRSCLLN--
---LA--PKEGLYNNCFVC--TTDE--N---------LIPCRV-----AIDSTHVH--IE
MD--------DIGTSH--LVG---------TQ--DRVN---VEFGDEVV-----------
---------------------------------------
>plant-like_FADS_Vaccinium_corymbosum
------------------------------------------------------------
--------GHR-ELAIQAAKVGVPFLLSFVGMAEVLGWEP--------------------
------------------------------------RAPIVAKCDRKRVLSSWAPYC-GD
TIPVEYELEFSRVRHLTPRQFVEM-LSKKLGVR-GVVA----------------------
--------------GENYRFGYKAAGDSSELVKLSEEY--GMGAYIINTV----------
------------------------------MDKNQD--YGSMSSSNLK-EQGQVSSTR--
VRLALA-KGDMKYVSELLGRQHRLMLMVKDQEWV--------ISNQHRVSARRSCFLN--
---LP--PKEGLYESCSVV--IGND--NN--------VMPCKV-----TIDASDIH--LE
LH--------DAATCI--RII---------SQ--DFQCL-GIDFG---------------
---------------------------------------
>plant-like_FADS_Populus_trichocarpa
-----------------------------------------------VAGG---IVALGK
FDALHI--GHR-ELAIQASKVGAPSLLSFVGMAEILGWEP--------------------
------------------------------------RAPIVAKCDRSRVLSSWAPYC-GN
LVPEEVQIEFSCVRHLTPRQFVEK-LSKELGVI-GVVA----------------------
--------------GENYRFGYKAAGDASELVRLCKEH--GMGAYIISSV----------
------------------------------MDNNQDYRSMN-SNDL-K-DRGQVSSTR--
VRQALA-VGDMKYVSELLGRHHRLILMLKDQEE-----RAKTSSG-WRVSAPKSYLLN--
---LP--PKDGFYENCSLL--FGN-KNPV----------TCRV-----FMDTTHIH--LE
TD--------EADPFD--FET---------DQ--EPHLL-GIEFGDSRPDRD--------
---------------------------------------
>plant-like_FADS_Oryza_sativa
-----------------------------------------------VIDG---IVALGK
```

```
FDALHI--GHR-ELAMYASKAGTPFLLSFVGIAEVLGWEY--------------------
------------------------------------RPPIVAQCDRKRVLTSWAPYC-KN
VVPIEYQVEFSKVRYLTPRQFVER-LSRDLKIQ-GVVA----------------------
--------------GENYRFGYRASGDAAELVKLCEEF--GLSAFIVRSV----------
------------------------------MDTAR-SYNGVTTSVNSS-DKGQVSSSR--
VRHALA-MGDMEYVSELLGRKHRLVLTVK----------ENHLQERKRIMLPKSCMLN--
---MP--PADGLYENCDLL--NGG-HLGL-----------CRV-----IINSETIV--IE
MKD-------ENSLL---PNT---------IQ--ENQQL-GIEFG---------------
---------------------------------------
>plant-like_FADS_Malus_x_domestica
--------RFSSILPSSKSSSPDITALPHCFSQREDECELPPEGLSSVAGG---IVALGK
FDALHI--GHR-ELAIQASKVGPPFLLSFVGMAEVLGWEP--------------------
------------------------------------RAPIVAKCDRKRVLSSWAPYC-GD
MAPAEFQIEFSSVRCLTPRQFVEK-LVKELGVR-GVVT----------------------
--------------GENYRFGYKAAGDATELVRLCEEY--GIEAYIINSV----------
------------------------------MDKNEY--SINIQWSDLKSDRGQVSSTR--
VRRALA-VGDMKYVSQLLGRQHRLIMNASKDQEGFP-------GCKHKLSVPKSSLLN--
---LA--PKEGLYEKCYLC--IGEDA-DR--------ASSCRV-----VVDTGYVH--IE
MDD-------EVDVCVCNRVG---------TH--DLRLLR-IEFG---------------
---------------------------------------
>plant-like_FADS_Glycine_max
IP----------------------LLFD-CFSQQEEDREILSDGTSAVAGG---IVALGK
FDALHI--GHR-ELAIQASRAGPPFLLSFVGMAKVLGWEP--------------------
------------------------------------RAPIVAKCDRKRILSSWVPYC-CN
MVPEEFEVEFSSVRHLNPRQFVEK-LSKELRVR-GVVA----------------------
--------------GENYRFGYKAAGDALELVKLCEEY--GMEAYIIKSV----------
------------------------------MDKN-RFSADMNSVTNSK-ERGQVSSTR--
VREALA-VGDLKYVSELLGRPHRLILMATDQERFS------TGQYK--VSAPRSCLLN--
---LA--PKEGLYEKCS-----LLLDQEN--------VVQCSV-----VIDSKFVH--IE
TD--------YGGLSD--IFC---------SQ--NLKFLF-IEFG---------------
---------------------------------------
>plant-like_FADS_Solanum_tuberosum
SCLSNKVCDFGVNCVPPKSSDTQSSLLSETLSQSGTDEEPPSERLPILTGG---IVALGK
FDALHI--GHA-SLRSSSXER-IXVLLSIVGNAGSYLNGN--------------------
-------------------------------GLQ-LLLNVIAKG-SFRLGLLIAVMCQGN
S--RI---DFSKVRYLTPCQFVEK-LSKELGVR-GVVA----------------------
--------------GENYRFGYRAAGDASDLVKLCEEY--GLEAYIINSV----------
------------------------------MDTNQISGALNSNDGKRE-QGGQVSSTR--
VRYALH-KGDMKYVSELLGRNHRLILMMEDQERF--------TSERNRLSAPKSCLLN--
---LA--PKEGLYENCSVL--IDKN------------VIPCRV-----IVDTTDIH--LE
CY--------EVDKFS--CVT---------SQ--DLKIL-GIDFG---------------
---------------------------------------
>plant-like_FADS_Ostreococcus_lucimarinus
-----------------------------------------------ATGERPLAVCLGK
FDAMQP--GHL-ALAERAATRGDVVVVSXSGMAETLGWET--------------------
------------------------------------RLPITAPSDRSRVLAMWSGTT-GA
AVSEH-ALAFGDVRELSPEAFVEM-LAR-MGVG-AVVT----------------------
--------------GKNYRFGYKAAGDVEALKRFGEAN--GLTVDVVDLVP---------
--------------------------------------ARRPSSAGL---GDQVSSSR--
IRSALS-AGDIDEVNEMLGREHRVVCDMTG-ERAANALRAAVDRNLDEINLSLESAEN--
---QP--PSDGAYSA---------------------------------------------
------------------------------------------------------------
---------------------------------------
>plant-like_FADS_Ananas_comosus
-----------------------------------------------------------K
FDALHI--GHR-QLAIHASKAGTPFLLSFIGMAEVLGWES--------------------
------------------------------------RPPIVAKCDRKRVLSSWAPYC-GN
VAPLEYQVEFSKVRYLTPRQFVER-LSKELRIS-GVVA----------------------
--------------GENYRFGYKASGDASELVRLCKEY--GLSAFIVSPV----------
------------------------------MDKANRSYNGATTSINSS-DKGQVSSTR--
VHHALA-TGDIDYVTELLGRKHRLMLSLN----------EGCLSMEKRILAPRSCMLN--
```

```
---QP--PGDGMYENCDFPVN--NELVGS-----------CRV-----AINAENID--IE
LND-------PSFWQE---NA---------TK--DGDII-GIEFG---------------
---------------------------------------
>plant-like_FADS_Micromonas_sp_RCC299
-----------------------------------------------EDGGK-IVVALGK
FDAMHR--GHA-ELARRASKMGAPILMSFGGMAEVLGWEE--------------------
------------------------------------KLPVVAPGDRARVLEMWKAAC-EG
KTVREHVIPFADIRRMSPEEFVST-L-KDIGVG-GVVA----------------------
--------------GANYRFGFKAAGTADILKDLGEKL--GVDVSIVDLL---PANEPG-
--------------------------------------DEGVYPAGYEN-TPQVSSTR--
VRACLA-AGDVEQAARLLGRNHALVLRIAG------------AGNDGR--FPTSAATN--
---QY--PAIGRYAAIV----VGADGG--------------RT-----PTTAEVTD--GE
VL--------EG-WGE--TNG---------GN--DNVC---VEFVGPAADA---------
---------------------------------------
>plant-like_FADS_Triticum_aestivum
-----------------------------------------------VLDG---IVALGK
FDALHI--GHR-ELAMHASKSGNPFLLSFVGMAEVLGWEY--------------------
------------------------------------RPPIVAHCDRKRVLSSWAPYC-RN
VVPLEYQVEFSKVRYLTPRQFVER-LSTDLRIK-GVVA----------------------
--------------GENYRFGYKASGDAAELVKLCEEF--GLSAFIVRSV----------
------------------------------MDTAKGSYNGAIPTVNSS-DKGQVSSSR--
VRQALS-VGDIEYVSKLLGRKHRLVLTVS----------EYSIKERKNIIVPKSCMVN--
---MP--PADGFYENCELFN--GG-YLGL-----------CRV-----XINTENID--IE
MKD-------ESSLS---PDT---------FQ--EGKQV-GIEFG---------------
---------------------------------------
>plant-like_FADS_Picea_sitchensis
PVRVSKILKGSLNVTKVRNSSGAQEANTETKVGQEKILIDCGPDQQCVLGG---IVALGK
FDALHV--GHR-ELAIRAAKIGVPFLLSFVGIAEVLGLEK--------------------
------------------------------------RLPVVAKCDRKRVLSLWAPLC-DG
VVPHEYHVQFAHVRYLSPRQFVEK-LSKELGVK-GVVA----------------------
--------------GANYRFGYKALGDASDLVQLCGEY--GLKAYIVDSV----------
------------------------------MDKFDGSSLEQENTGTDLREKGQVSSTL--
VRKALA-AGNIKRVEQLLGRKHRLMLTTDN-----------CIVTKNIFVSCRSSVLN--
---QP--PREGQYG-CMIMIK----------------DDVDRN-----SNGNENIIGYGD
LKIN---SQIEVTLHEG-SFG---------AHL-NGKSFIDLEFEGSREC----------
---------------------------------------
>plant-like_FADS_Mimulus_guttatus
SSFVTNNNNNEDLVSSINKEVCTQDILSDSLSRAENDENSLSEGL-AVAGG---IVALGK
FDALHI--GHR-ELAIQAAKIGVPFLLSFVGMAEILGWEP--------------------
------------------------------------RAPIVAKCDRKRVLSSWAPLC-GN
IVPKEFQIDFSKVRSLSPQQFVEK-LAEELGVA-GVVA----------------------
--------------GQNYRFGYRAAGDASDLVRLCNEY--GMRASIINSV----------
------------------------------MDNNQESVNETFSS-NSH-EKGQVSSTR--
VRHALA-KGDMKYVTELLGRHHRLIVRVKNSENVF------RERKRLLV--PRSCLLN--
---LA--PKEGLYENCSLV--IGDDDQNV--------VVPCRL-----VIDTTDIH--LE
LLD-------ELAPPI--NTN---------TQY-FESL--GIEFSDSKV-----------
---------------------------------------
>plant-like_FADS_Gossypium_raimondii
IP-----------------------FPPVSFSQREDEHEKPSEALSSVAGR---IVALGK
FDALHI--GHR-ELAIQASKVGTPYLLSFVGMAEVLGWDP--------------------
------------------------------------RPPVVAQCDRERVLSSWAPYC-GN
VAPKEFQVQFMSVRHLSPRQFVEK-LARELGVC-GVVA----------------------
--------------GENYRFGYKAAGDASELVRLCDEF--GMGAYIINSV----------
------------------------------MDRHQDAR--NMNRLDLK-DRGQVSSTR--
VRQALA-EGDMKYVSELLGRPHRLLLTIKDWESLT------STSSTQRMSAPRSSLLN--
---LP--PKDGFYENC-----SLLFGEQN--------AVTCRV-----SIDTSHIH--LE
MD--------RVDFCD--NDY---------SQ--KSQVL-GIEFG---------------
---------------------------------------
>plant-like_FADS_Brachypodium_distachyon
-----------------------------------------------ISDG---IVALGK
FDALHI--GHR-ELATHASRSGNPFLLSFVGMAEVLGWEY--------------------
```

------------------------------------RPPIVARSDRERVLSSWAPYC-RN
VVPLEYQVEFSKVRYLTPRQFVER-LSTDLRIK-GVVAT---------------------
------------MTGENYRFGYKASGDAAELVKLCEEF--GLSAFIVRSV----------
------------------------------MDTAKRSYNGATPPVNST-DKGQVSSSR--
VRHALS-VGDMEYVSKLLGRKHRLVLTVN----------ECCLQEREKIVFPKSCMLN--
---MP--PADGFYENCGLVN--GG-YLGL-----------CRV-----TINSETID--IE
LED-------KNSLT---SNL---------FR--EDQQL-GIEFG---------------

>plant-like_FADS_Brassica_napus
TP-----------------------IIHSCFSQREDDLELPADGSTPVSGG---IVALGK
FDALHV--GHR-ELAIQASRVGTPYLLSFVGMAEVFGWEP--------------------
------------------------------------RAPIVAKCDRKRVLTSWDSYC-GN
KAPVEYEIEFASVRHLTPRQFVEK-LSKELRVC-GVVA----------------------
--------------GENYRFGYKASGDASELVRLCEEY--GIGAYIITSV----------
------------------------------MDKKQDSE--KRDSGDSK-DRGQVSSTR--
VRQALA-AGDMSYVSELLGRAHRLILQVNNTG---------DMQSERRISVPRSSVLN--
---LP--PGNGVYNAC-----LVLADDEP--------SVPCSV-----VVDSSYIH--VE
TE--------ELLLCN--SD--

>plant-like_FADS_Solanum_lycopersicum
SCLSNKVCDFGVNCVQPKSSDTQSSLLSETLSQSGTDEEPPSERLPILTGG---IVALGK
FDALHI--GHR-ELAIQAAKRGIPFLLSFVGMAEVLGWEP--------------------
------------------------------------RAPIVAECDRKRILSSWAPYC-GN
VMPKETQIDFSKVRYLTPCQFVEK-LSKELGVR-GVVA----------------------
--------------GENYRFGYRAAGDASDLVKLCEEY--GLEAYIINSV----------
------------------------------MDTNQISGDLNSKDGK---ERGQVSSTR--
VRYALHSKGDMKYVSELLGRNHRLILMMEDQERF--------TSERNRLSAPKSCLLN--
---LA--PKEGLYENCSVL--IDKS------------VIPCRV-----IVDTTYIH--LE
SY--------EVASFS--CVT---------SQ--DLKIL-GIDFG---------------

>plant-like_FADS_Chlamydomonas_reinhardtii
---MGAVLGW
PDRLPL--TAPQDRGRVLAGWAAALRSAASAVGSANGATS--------------------
------------------------------------RPSTEGNNGNGGTGTGAGDCG-AG
RVVRLRTMPFGQIRGMSPEEFVAL-LARDLGAA-GVVA----------------------
--------------GRNYRFGFKAAGDAGALVRLGAQY--GLKVSIVDLVSLEAAADSGG
SSDPASARASGSDGVGMSGTGTAAGNSSSGVAGASSSNDSNQASKAVA-ETR-VSSSR--
IRGLLE-GGHVEAVETLLGRKYRLVCDLTAAAATAGSADAAAALGTGLLRVPAACYRN--
---QA--PAPGQYRVMVRLVDSGPPGAEIAAERDSGGGAFMRT-----SLSSTFSSG-LD
PGAV------EPVVGPV-CVP---------VR---ISEE-GLEVDLGPPGVHG-------
---SDSPPP-----TRPLLLLDFD------------ACS
>plant-like_FADS_Citrus_sinensi

----------------------------FIGMAEVFGWEP--------------------
------------------------------------XAPIVAKCDRKRVLSSWAPYC-GN
VAPVEFQIQFSSVRHLSPQQFVEK-LSRELGVR-GVVA----------------------
--------------GENYRFGYKAAGDASKLVRLCEEY--GMDACIINSV----------
------------------------------MDKHQD--SRDIDCNDSK-ERGQVSSTR--
VRQALA-MGDMKYVSELLGRQHRLILTVNDHDELIS------TSNKHRVSVPKSCLLN--
---LP--PKEGFYGNSVLL--FGEE--N---------PVKCKI-----CIDASHIH--LX
MD--------KVXFCN--FDH---------SQ--EL-VL-GIEFG---------------

>plant-like_FADS_Physcomitrella_patents
---VAGG---VVALGK
FDALHV--GHR-ALAEHAAEIGAPFLVSFAGMAEVLGWEV--------------------
------------------------------------RLPVVARCDRARVMSLWAEHC-GD
VVPQEYMLEFSKVRSLSPEQFVEK-LASELKVK-GVVA----------------------
--------------GANYRFGYKAAGDASDLVRLCQEY--GLQSAIVNPI----------
------------------------------MDASEMSSPVLVDDSS-R-EKGQVSTTR--
VRKALA-NGDMKRVAELLGRRHRLVIRPD-----------KYVRRDNLISVPAVNALN--
---QP--PRIGSYE-CSFVL-EGA-GATLDSDS---LAGNVRL------EES-CIT--LE

```
LQ--------QPSLVD--TIT---------QQ--NLIV---LDFLA--------------
---------------------------------------
>plant-like_FADS_Ricinus_communis
-----------------------------------------------LAGG---IVALGK
FDALHI--GHR-ELAIQASKVGTPYLLSFVGMAEVFGWEP--------------------
------------------------------------RAPIVAKCDRNRVLASWAQYC-DN
ITPLEFQIQFSSVRHLNPQQFVEK-LSKELRVC-GVVA----------------------
--------------GENYRFGYKAAGDASDLVRLCEEY--GLGAYIINSV----------
------------------------------MDNKKDPINSN-SRDS-K-DRGQVSSTR--
VRQALA-LGDMKYVSKLLGRRHRLMLMLKDQRG-----LTSSSSSRWRISAPKSCLLN--
---LP--PKDGFYKNCFLL--FGN-ENRM----------TCSA-----IIDSMYIH--LE
MD--------ELGLSI--YNG---------SQ--DFQLL-GVEFGD--------------
---------------------------------------
>plant-like_FADS_Manihot_esculenta
IP-----------------------IVSDCFSQEEDDHQIPSEGLSPVAGG---IVALGK
FDALHI--GHR-ELAIQASKVGSPYLLSFVGIAEVLGWEP--------------------
------------------------------------RAPVVAKCDRSRVLTSWAPYC-GN
IAPSEFHIEFSSVRHLSPQQFVEK-LSKELRVC-GVVA----------------------
--------------GENYRFGYKAAGDASELLRLCEEY--GMGAYIINSV----------
------------------------------MDKNKDSG--IIDLNDLK-DRGQVSSTR--
VRHALA-LGDMKYVSELLGRRHRLMLMLKEEKGFT------NSSGRWKASIPKSCLLN--
---LP--PKDGFYENC-----FLLFCDEN--------MVKCSV-----FIDSTFIH--LE
TD--------EVGLNN--FSV---------SQ--DFQLL-GVEFGH--------------
---------------------------------------
```

Figure S3.- Trimmed multiple alignment of the set of FADS-type I, FADS-type II and plant-like FADS protein sequences used to build the PHYML maximum likelihood tree in Figure 3 of the paper. The alignment was trimmed with the ‘automated1’ option of the trimAl software.

```
>OUTGROUP_AtFHy/RFK_Arabidopsis_thaliana 312 bp
----EFYPLFSAQMDKIKSLPGANRLIRHLKCH-------------------GVPVALA
SNSSRANI--KISYHEGWKECFSVIVG-------SDEV-SKG---K-PSPDIFLEAAKRL
KKDPA-DCLVIEDSVPGVMAGKAAGTKVIAVPSLPKQTHLYTSADE-------------
--LLDIR-LEKWGLP-PFQDWIENTLPIDPWHIKGFGRG-S--KVLGIPTANLDELVEHP
SGVYFGWAGLMVMSIGWNPY-IEPWLLHDFYGE--ELRLVGYIRPEANFSS-LESLIAKI
HED----REVAE
>FADS-type_I_Thermotoga_maritima 312 bp
------MVVSIGVFDGVHI--GHQKVLRTMKEIAFFR--------------SLIYTISY
PPEYFLPD---GLLMTVESRVEMLSRY--ARTVVLDFF--RI---KDLTPEGFVERYLSG
---VS-AVVVGRDFRFGKNASGNASFLRKK------GVEVYEIEDV-------------
GKRVSSS-LIRNLVQ-EGRVEEIPAYLGRYFEIKDREFG----RKLGFPTANILVD--LK
RGVYLVRVHL-VMNVGFRPTKYEVYILDDLYGQ--RLKLLKFMRDEKKFDS-IEELKAAI
DQDVKSARNMID
>FADS-type_I_Corynebacterium_ammoniagenes 312 bp
------SAVTIGVFDGVHR--GHQKLINATVEKAREV--------------AIMVTFDP
HPVSVFLPAPLGITTLAERFALAESFG--IDGVLVIDFTREL---SGTSPEKYVEFLLED
TLHAS-HVVVGANFTFGENAAGTADSLRQICQSR---LTVDVIDLL------------E
GVR-ISSTTVREFLS-EGDVARANWALGRHFYVRGAGRG-G--KELGFPTANQAL---PA
DGVYAGWLTIAAISVGTNPT-VESFVLDDLYGH--DVKVVDHVRAMEKFDS-VEQLLEVM
AKDVQKTRTLLA
>FADS_type_I_Paulinella_chromatophora 312 bp
------TAVAVGSFDGLHK--GHRRVITNISENSTTI--------------PTVVSFWP
HPREVLYG-RLRLDMPAEKLTLLESLG--IKQLVLVPFSERL---AELTPEIFVRQVLKQ
QLGAL-KVAVGKNFRFGVNRSGDTSALGRIAQEM--GIKVEILPIL-------------
--RVSSS-RIRRALG-EGKIQEATRLLGRPYRFNSSDVK----RNHGLPTLKVFL---PR
QGIYAVWVRLSGGPIGAIMS-VEVYFLDDFDEV--RVYIVSLLRGQQEFWG-TEGFYQQI
HNDIMQARKRLE
>FADS-type_I_Micrococcus_luteus_NCTC_2665 312 bp
------TVVTLGNFDGVHR--GHREVLRRVVELARAR--------------AVAVTFTP
HPRAVHQPPHVDIISPEQRVVLLEEAG--LDAVLLQRYTLEF---ADQSPEEFVRGMLVH
GLHAA-VVVVGHDVRFGRGNTGDVAEMVRLGAHY--GFEVEAVEEF-------------
PERRCSSTWVREALD-AGDVAQAAAVLGRHHVLHGFARG----RELGFPTANLMI---PA
DGVYAGWVHDAAISIGSNPT--EAHVIDDLYGQ--HIEVVARLRGMVAYEG-VEKLVAQI
TQDVDEARAILA
>FADS-type_I_Desulfomicrobium_baculatum_DSM_4028 312 bp
-------CVTIGNFDGVHI--GHQRLIARVRDLAAGF--------------SVVITFEP
HPLRFFTGTPPFITLYEQRAELIRSLG--IDHLLCLEFNQAL---ASMSPEDFVRRILVE
GLHIK-ELVIGYDYAFGKGRRGNYALLSQLGKQW--AFG---VEQL------------Q
AIVSSTR--IRDLVE-AGDVWAAKPLLGRFYRVHGQNRG-G--RLLGFPTANVLF---PK
TGVYCCWAELAVANIGYNPTSVEVHVMDDLYER--TLKVVQRLRGERKFSG-LDELKAQI
GKDVALARTILA
>FADS-type_I_Mycobacterium_marinum_M 312 bp
------CVLTVGVFDGVHR--GHAELIAHAVKAGRAR--------------TVMMTFDP
HPMEVVYPHPAQLTTLTRRAELVEELG--IDVFLVMPFTTDF---MKLTPDRFIHELLVE
HLHVV-EVVVGENFTFGKKAAGSVDTLRHAGERF--GFAVEAMSLV-------------
-TVTFSSTYIRSCVD-AGDVMAATEALGRPHRVRGYGRG----AELGFPTANVAI---PA
DGVYAAWFTVAAVSVGTNPT-VEAFVLDDLYGQ--HVALVARIRGQKKFAS-VPELVAEI
GADTERTRVLLS
>FADS-type_I_Chlorobium_chlorochromatii_CaD3 312 bp
------SVVTVGSYDGVHC--GHRVILSRLVEVAHHN--------------SVVVTFEP
HPRTVLKGPLGLLTTLEEKSDLLAAAA--VDLLFVVRFTHDF---AARTSDDFIRNVLVG
LLGAE-RIIVGYDHAFGRDRSGSHNTLERLGNEL--HFGVEVIDEV-------------
```

--LSSTR--IRKLLQ-DGRIEEVNEFLGSPYLIQGAQLG----RTIGFPTVNLLL---PR
YGVYFARTMVALMNIGKRPTTIEAHLLGSLYGE--ELRFLRFIRDEKRFAS-LEALQEQL
EKDKKAVEMYLE
>FADS-type_I_Chlamydia_trachomatis_D_UW-3_CX 312 bp
------ESVTIGFFDGCHL--GHQALLSFLTKFPSK---------------SGVITFSQ
HPEHTLSN-PETITSLEERVQLLAGCG--IDYLAVLPFNQEI---ANQEAEPFIQSIYKT
-LRPS-RIVLGYDSRLGKGGLGTAQTLRPFAASL--GISLEEVPPLT-------------
-IVSSRK--IRQFLR-KKDLCSAEKFLGRPFSYHGRGIG----ASFGYATINLLL---PL
-GVYTCTIV-GVMNLGMAPTCLEAHILDDLYDK--SITVEQFLREEKLFSS-KDELVLAI
QEDIRQARLNKN
>FADS-type_I_Bacillus_subtilis_subsp_subtilis_str_168 312 bp
------SVMALGYFDGVHL--GHQKVIGTAKQIAEEK---------------LAVMTFHP
HPSHVLGRPKDLITPLEDKINQIEQLG--TEVLYVVKFNEVF---ASLSPKQFIDQYIIG
-LNVQ-HAVAGFDFTYGKYGKGTMKTMPDDLDGK---AGCTMVE-KDK------------
-KISSSY--IRTALQ-NGDVELANVLLGQPYFIHGDKRG----RTIGFPTANVIVP--PT
-GVYAVKAEVGVCNIGYKPTSIEVNLFDEVYGA--AIKIYKRIRSERKFNG-IKELTEQI
EKDKQEAIRYFS
>FADS-type_I_Clostridium_thermocellum_ATCC_27405 312 bp
------TGVGLGNFDGLHV--GHMALIDKLIKESESN---------------SVVYTFSK
HPENIIRKFTPLITSTRKKVELLEKTR--LDYLYFEKFDETF---SRMRPEDFVKDILVE
RLNMK-LAVAGFNYRFGYRGMGDTELLRKLGRKY--GFRVIVIEPV--------------
-VVSSTK--IRNYIL-EGDMERVFAFLGRHYSVKGRRVG----NTIGFPTANIVL---PC
HGVYITRT--SITNVGNNPTSVETYIFDDIYGK--EIEVISKIREEMRFGS-VEELIEQI
HKDIEIAKKVLA
>FADS-type_I_Lactobacillus_fermentum_IFO_3956 312 bp
------VVVAMGFFDGVHR--GHQAVIARAKEEAVRR---------------LAVLTYDK
LPGIVYQRGVHYLTTIDRKLALLEQLG--ADLVYLVDFTAKL---GSLSPEEFVSDYLVR
-MHAV-AVVAGFDHTYGKKDVATMDRLVGYAAGR---FDVVTVK-KGA------------
-KIGSSR--IRDLID-QGRVEDANQLLGYRYQTHGLARG----RTIGFPTANVRI---PA
VGVYTVNFLVGMASVGYNVTTIEVYLFDNIYGE--HVTVIQRLRGEIKFDG-VDGLIDQL
HADQINSLAILR
>FADS-type_I_Mycoplasma_penetrans_HF-2 312 bp
------FNNQIENTENLVL--GHFNLIHYGHHELFKE---------------FSFLIFEN
NPSKFKR-----PYSLDERIENLSKFN--PEYIFVYDILKNN---I--DADVFIKEVLLK
-IKPK-NIVVGSDFCFGKNKKGNVELLKS-------FFNLKEIYKN--------------
----SSRN-IIELIE-SGFLEKANEMMMFNFYYKGKGLA----SELDVPTANIKI---PS
-GSYSSITL-SISFIGIPKSTVETHIFDDIYNK--KIKIIKFIRPNQKFDD-IKTLIKFI
KNDCDIAKKFLS
>FADS-type_I_Chloroflexus_aggregans_DSM_9485 312 bp
------TVLTIGRFDGVHL--GHQQLIRTTVERARAL---------------SAVLTWEP
NPRAVLQPPLQLLTDLDEKIEQIRRLE--PDLLIIAPFTNEI---RQLSAAEYMARICAA
-VPVR-EIWVGEDFAMGRGREGDIPRLMEIGREL--GFA---VGALMA-----------G
IPVSSSR--IRELVL-AGNVAGAGALLGRPFALRGDGRG----RQIGFPTANLVV---PA
HGVYACRTYLTVTNIGVRPTVVEAHLIDDLYDQ--HLRILMRLRNERKFSG-IDELVAQI
RHDVAEARVVLG
>FADS-type_I_Rhodospirillum_centenum_SW 312 bp
------CAVALGNFDGVHR--GHRAVIAATQEKAAAL---------------TVVLTFEP
HPRSLFRPPPFRLTPFRIKARLIEALG--VDALFVIHFDEAF---SRRTAEEFVEHVLIQ
ELGAA-HVVAGYDFVFGHKRGGDMALLRRMGEER--GFGVTEVRPVGT------------
-VFSSTR--VRELLQ-AGEPQAASGILGHPFELHGDKRG----RTIGFPTANVLR---PR
FGVYAVLAGIGVANLGRRPTRLEVHLFDDLYGR--HLRVLHFLRPELKFDG-LDALKAQI
AADALAARGLLA
>FADS-type_I_Desulfobacterium_autotrophicum_HRM2 312 bp
-------VITIGNFDGVHK--GHQAIFHQVIEKAEEI---------------SVVMTFDP
HPLKVL-GGPPLITRKDQKIELISATG--IDKILCLPFTREF---AAISAQEFIKDLLIN
QLGMK-AIVVGLDYSFGRNREGNLELMQKAGKQL--GFEVLIADWITG-----------S
ERISSTR--IRELVM-EGRVDETPKFLGRFYQIKGRQRG-G--SKLGFPTANILS---PK
MGVYAVTVETGVANIGYSPTTIEVHLLDDLYNT--RIRIIKRLRDEKKFSN-LEELSNQI
RNDIQLAREILA
>FADS-type_II_Alistipes_putredinis_DSM_17216 312 bp
-------AATVGSYDGVHS--GHRVLLDRIRREAAAVG--------------SIVLTFAP

HPRVTLGT-LRLLTSLEEKIYLLDRFG--IDNLIVIPFDRAF---SRIPSESFVKDYLIG
KVGVK-NLVVGFNHRFGHDKEGDYRLLNGLHDEF--GFRVTEIEKQEK------------
--VSSTV--IRRLIE-RGEMNKAARMLSHPYLL--------------CAGHIALL---PP
PGEYPVRIEGGVLRIG----TPELLRTA-------PSGHIEF------------------

>FADS-type_II_Eubacterium_saphenum_ATCC_49989 312 bp
------RAVALGNFDGVHI--AHVKLIDMIKKRARAF---------------ACVFTFKN
HPKEFIPGILKYINDTEKTMEIFENLG--IDSLVMVPFDEVL---QRMSPEDFVKDVIVE
RLGAK-YVCVGYDYRFGFEGKGDVSLLDRLGKTY--GFHVDVIDKVV-------------
-TVSSSR--IRKLIY-EGDFEGVQKLLGRRYMIHGKSIG----RKLGFRTLNICI---PS
DGVYVTRTRVSITNIGTAPTSIETNVFDDIYGD--IVHILKKIRDEIKFDT-PEELSKQI
AEDVRLAKDIHS
>FADS-type_II_Arthrobacter_chlorophenolicus_A6 312 bp
------TVVSLGCFDGVHR--GHQLLLARLLNAAETN---------------SVVVTFDP
LPAQLLYPAPLAVMALEDRLAALEVEG--VDAVLVIRYTRDV---AAQAAEVFVEKVFVK
TLHAA-AVVVGEDCRFGQGRAGNIETLRAAGLRW--SFDVSVLGDR--------------
-GRRYSSTWVREVLS-SGDVGIAEQILGRPHRIRAAGSW----RTK-------ML---PA
SGDYAGWARVAIIRIRASLATVELQMPAKS-PG--MSRKFDFSSTS--------------

>FADS-type_I_Arthrobacter_chlorophenolicus_A6 312 bp
------SVVTFGNFDGVHR--GHQQVLSQLIRSARLT---------------AVAVTFDP
HPAVIHRPAPRLIMGLEDKLEALGELG--LDAILVVKYSLDL---ASLTAEEFVEQYLVD
CLHAS-HVVIGHDARFGRGNSGDLETMKALGGKF--GFEVQVISEFYPL--HD-------
--RRCSSTWVREALQ-EGDVATAAEVLGRAHRMHGAARG----RALGFPTANLLI---PA
DGIYAGWLVDAAISVGSNPTQVEAHVIDDF--------DGQTVIVEFVARLRGMVAYRG-
PEALVDQQA---
>FADS-type_II_Lactobacillus_plantarum_JDM1 312 bp
------LVLALGFFDGVHQ--GHQRVIQTAKRIAKQR---------------LAVMTFNR
HASQLFKSTFRYLNTVAQKSQHMAALQ--VDRLYITDFNHQF---AGLTPTAFIKDYLVG
-LNAQ-VVVAGFDYTFGQGGANGMRELAELGAPY---FETVTVDRLLK------------
--VSSTR--IRGLIA-RGQIEQANDLLGYTYATPLT-------RTIQLANRQQ-----PA
AGDYRCWLVSVILRVTTTLKPPETSVLDDVQWQ--QRALVSSVSAQHQQS--------QY
SKA---------
>FADS-type_I_Lactobacillus_plantarum_JDM1 312 bp
------IVLALGFFDGVHR--GHQQVVATARQAAQAQ---------------LAVMTFDQ
HPSVVFKHQVRYLTTIDQKTALMSELG--VDILYVLHFDATV---GAMPPQTFVDQLIVG
-LHAQ-TVVAGFDYTYGPAEIANMQRLNDYGHNR---FEIIEVP-KAE------------
-KISSTR--IRRALD-AGDIDTANRLLGYQYETHGEARG----RTLGFPTANVRV---PG
IGIYATMVQIGMASVGRNVTTVEIYLLDDLYGH--NLTVGHRMRGEIKFAG-ADALVAQL
KRDEQNTHPFVV
>FADS-type_II_Listeria_monocytogenes_HCC23 312 bp
------AVLTIGKFDGVHI--GHQTILNTALSIKKEN---------------LTAISFSP
HPLWALKQYREMLTPRMEKERWLAHYG--VDHLIETAFTPRY---AETTPEEFVRDHLTN
-LNLS-HIVVGSEFNFGKGRDSDVDLLRDLCKPY--DIGVTSVPVITK------------
--ISSTN--IRAFIR-RGHFQEAEQLLGHPWYINGEMIG--------------VL---PA
TGTYQTDS--GMVNV-----LVELSDGLS-------------------------------

>FADS-type_I_Listeria_monocytogenes_HCC23 312 bp
------KVMALGFFDGVHL--GHQAVIKKAKQIAEQK---------------TAVLTFDP
HPSVVLSNQVKYLTPLEDKAEKMAELG--VDIMYVVRFTTQF---SELSPQSFVDNYLVA
-LNVE-HVVAGFDYSYGKKGEGKMTDLAQYADGR---FEVTIVD-KSD------------
-KISSTN--IRRAIT-EGELEEANQLLGYPYTTHGDKRG----RTIGFPTANILI---PK
LGVYAVKFRVGMASIGYNITSIEVYILDAEIEW--YQFFELKFNG-VEGLIAQLEKDEQD
T------RAYFA
>FADS-type_II_Treponema_denticola_ATCC_35405 312 bp
FPEGKETAISVGGFDGPHK--GHDKLLRQVLTYA----A-------------PGLVTFFR
SPAAVKNK-SGDVSSLRLRLKKFQELG--FHFIVLIDFSASF---AKIEGTAFFD-ILIK
TIRMK-YLAVGSDFFCGYRRGLGVDDLKKIAPQK--GFCFDSIDPVLK------------
--ISSSA--IREAVR-LGDFSLAKELLGYPFLFPWEVK---------------IFA--PK
--AYISQILPLVQKT-----RQEQE---D--GL--LLCF---------------EKD-SK
AFDLKD-TIEFI

```
>FADS-type_I_Treponema_denticola_ATCC_35405 312 bp
------SCVAVGFFDGLHV--GHRAVIDRLCKCKNLR--------------PVLISLSN
NSSPVIY-------TEEEKSYLLQ-NGK-LDTMFSLS-EDII---KNMTAESFAHDVLNK
MLNTK-TLVAGENALFG-LDQVDVNHFRSIGKKY--GFTVETVPME-------------
--VSSNT--IKQTIH-DGDFSKVSSMLGSSYLIHGKGAG----HKFGMPTANILF---PP
HGVYGSISRFGMTNIGLRPSTIETFLLNDIYGQ--KVFLLVYIRGIRKFDGGLAEVRQQI
DKDIKQINGNLL
>FADS-type_II_Treponema_pallidum_subsp_pallidum_str_Nichols 312 bp
----RGAAISVGGFDGPHR--GHAFLFDKVFAAACAPVA-------------TGLITFT-
HPPRKHKTYEGDLSTLRLRLRYFRARG--FDFVVLIDFSKDF---ARIPGGVFFN-TLLR
AVRVC-YLAVGVDFRCGHGLDTGVRELRRLGDAH--SFVCDAVGHY-------------
--VSSSA--VRRAVR-CADFESARRLLGRAFSLPWQQSG-GCARTLCAERGRV-----PP
EGEYAVRLVQ-----GVGLRGTEALSHGEFE----------------------------
------------
>FADS-type_I_Synechococcus_sp_JA-3-3Ab_CYA_2407 312 bp
------TAVAIGNFDGLHL--GHQKVLQPIRDS-------------LQGVR--VLTFHP
HPQEVLTG-QLLLTPPAEKLALLAQMG--FEQVVLLPFTPAF---ARQPPQEFIQTVLEQ
GLRVR-HLSVGWDFCFGHRRSGNAQTLQAWGSER--CIPVEVIPEA-------------
-RVSSSR--IRAALA-TGEVAAATELLGRPYRLPGDRRG----RELGFPTANLFLP---R
DGVYSVWVNVGVMNIGHRPT-VEVHLLDDLYGQ--EVQVQGFIRPERRFPS-VADLIQQI
RQDCQTARQQLG
>FADS-type_I_Gloeobacter_violaceus_PCC_7421 312 bp
------CAVALGNFDGVHL--GHQAVIQAVLG--------------RAGIP--VLTFDP
HPREYFTG--FLLAPERERTAAILALG--IAQVLVLPFDELL---AATEAGAFVEQVLVA
GLGAR-FVSVGWNFRFGKERAGTTEMLQSYARAG--AFDIEILAER-------------
-RVSSSV--IREALG-CGDLDLARLLLGRAYGLRGDQRG----RLLGFPTANLFLP---K
DGVYLVSARW-LLNLGLRPT-IEVHVLDDLYGQ--HIKIERYLRPEQKFGS-PGELVAQL
HRDREAAKEIAG
>FADS-type_I_Thermosynechococcus_elongatus_BP-1 312 bp
------TAIALGNFDGVHR--GHQEVIRTLLKAAP------------PDCYP--VMTFSP
HPQAFFTG-RLLLTPEAEKRALLHQCG--IAQVIVLPFTQAL---AQLSPLEFVEQILVQ
QLQAK-VLSVGFNFGFGRGRSGTAEDLRSLCAPF--GIVVHIVPPY-------------
-RVSSSA--VRAALA-AGEVALARELLGRAYTLQGEQLG----RQLGFPTANLLL---PR
YGVYACRVSGGVVNIGVRPT-TEVHLLHNLYNQ--EITLEAFIRPELTLPS-LAALRAQI
AADCQVAVDLLE
>FADS-type_I_Acaryochloris_marina_MBIC11017 312 bp
------TVIALGNFDGIHQ--GHCQVIQSLLSNR-------------SDTAQ-TVVAFNP
HPQAFFSG-QPLLTPLSEKATLLENLG--VDQLVLIPFDQAL---ANLSPQDFVKDILVA
QLQAQ-FISVGFNFCFGYQRAGTAEDLVAIAGQY--QIPVS-ITAP-------------
-PISSSA--IREALL-EGNLEKAQQMLGRAYDLKGQQLG----RTLGFPTANLFC---PR
TGVYSVSVTSGVMNWGCRPT-LEVHLLDDLYGH--TVTVQQFLRSEQKFAS-LADLKAQI
QADCEIARASLA
>FADS-type_I_Synechocystis_sp_PCC_6803 312 bp
------TAIALGNFDGVHR--GHGVVLRQVMDFAQAV----------EHLLH-AVVSFNP
HPRSFFSG-QPLLTPLPEKAAQLTAIG--IEQLVLLPFTEKL---ANLSPKQFVQSILVE
QLQAK-FISVGQDFCFGHQRRGNVQDLQNLGQEF--GITVAIAQLE-------------
-RISSSR--IRRALK-EGKLAMANHLLGRPYALQGQQLG----RKLGFPTANLLW---PK
YGVYAGWVNLAVINLGDRPT-AEVHLLQDLYGQ--GLEVTHYLRPETKFAG-LDQLKNQI
AQDCQQAEKLLN
>FADS-type_I_Trichodesmium_erythraeum_IMS101 312 bp
------TAVALGNFDGLHQ--GHRQVIEPILNLKGRSKLLSVCPSELTRTTESTVVTFNP
HPQEFFSG-KKLLAPLEEKLAIFKHIG--VEQVVLLPFDRNL---ADLTPNKFVEEILVK
GLQVH-QISVGCDFCFGQKRAGKAKDLQAIAGYY--DIDVTTVPLY-------------
-RISSSI--IRQALE-KGDLDKSNRLLGRPYSLHGQQLG----RTLGFPTANLFL---PR
FGVYAVEVYLGVMNVGCRPT-VEVHLLDDLYGQ--TLTVIEFLRPEQKFAS-LDLLKAQI
YEDCRIARSILK
>FADS-type_I_Prochlorococcus_marinus_str_NATL1A 312 bp
------TALALGSFDGLHL--GHKKVINAILK-------------EPIGVP--VVSFWP
HPREVLFG-RLRLDLPNEKTFLLEPLG--IEQLVLVPFNKNL---ASKSAETFVEEVLVK
TLHAK-HIAVGENFRFGRNREGDTSTLKKIGTSL--GIKISIVPIV-------------
-RLSSSR--VRKALN-DGDLKHAKHLLERPYTFKGRGLG----KKIGWPTANLFLP---S
```

LGVYAAWASIAVMNMGPQPT-VEVHLLDNLLGH--ELIIVQRIRLQKKFDN-IESLSKQI
SSDAKLAKEILT
>FADS-type_I_Nostoc_punctiforme_ATCC29133 312 bp
------TAVALGKFDGVHL--GHQRVIQPVLHPCDRLS-----VASSPQSKESTVVTFDP
HPQEFFTG-RTLLTPLDEKVQQLRSLG--VEQLVLLPFDKEL---SALTPEEFVQKILVQ
QLQCQ-RISIGQDFCFGEKRSGTAKDLQLIAAKH--NIPVTIVPLQDSPTQSS---T-QD
ARISTSL--IRQTLE-QGDIENANLLLGRPYTLQGQQLG----RTIGFPTANLFL---PR
QGVYAVRVFTGVMNIGNRPT-AEVHLFDDLYGK--KLAIVKFLRPEQKFPS-LEALKTQI
QLDCVVAKEVLS
>plant-like_FADS_Arabidospsis_thaliana_AtRibF1 312 bp
VAGG---IVALGKFDALHI--GHR-ELAIQAARIGTPYLLSFVGLAEVLGWK--------
--------RAPIVAKCDRKRVLSSWASYC-GNIAPVEFEIEFASVRHLNPQQFVEK-LSR
ELRVC-GVVAGENYRFGYRASGDASELVRLCKDF--GISAYIINSVMDKNQVSDSKSKER
GQVSSTR--VRHALA-AGDVRYVTELLGRPHRV------TSKRGRISLQTSSL-LP--PG
NGVYKACSLI-SCKVIVDTS--EERFHN---SQ--EFQLIEFG-----------------

>plant-like_FADS_Ipomoea_nil 312 bp
VAGG---IVALGKFDALHI--GHR-ELAIQAAKIGVPFLLSFVGMAEVLGWE--------
--------RVPIVAKCDRKRVLSSWAPYC-GGMIPREFQIEFSKVRYLTPRDFVEK-LSK
DLGVL-GVVAGENYRFGYKASGDASDLLRLCNEY--GIQACIINSVMDKNQDP-DAK-ER
GQVSSTR--VRYALA-KGDMKYVSELLGRHHRLDQEKFT-SDRCRVSAPKSCL-LS--PR
EGVYENCSIMIACRVVIDTT---KHTC----CQ--QLNLID------------------

>plant-like_FADS_Zea_mays 312 bp
VLGG---IVALGKFDALHI--GHR-ELAMHASKAGTPFLLSFVGMAEVLGWT--------
--------RPPIVAHCDRKRVLSSWAPYC-RNVVPLEYQVEFSKVRSLSPRQFVER-LSK
DLRIK-GVVAGENYRFGYKASGDATELVKLCEEF--GLSAFIVRSVMDTAKRSINSS-DK
GQVSSSR--VRHALA-MGDMEYVSELLGRKHRL------LHEKKSIVLPNSCM-MP--PA
EGLYENCDLV--CRVIINSD--DNSLS----IQ--E-VQIEFG-----------------

>plant-like_FADS_Medicago_truncatula 312 bp
VAGG---IVALGKFDALHI--GHR-ELAIQASRAGPPFLLSFVGMAKVFGWE--------
--------RAPIVAKCDRKRVLSSWFPYC-GNMVPEEFEIEFSSVRHLSPRQFVEK-LAK
ELRVR-GVVAGENYRFGYKAAGDASELQKLCEEY--EMEAYIIKSVMDKN-QYIGSK-ER
GQVSSTR--VREALA-VGDMRYVSELLGRQHRLDRETFSSQYK--VSAPKSCL-LA--PK
EGLYEKCS--MLCRVIIDSK--YGGSSD---TH--DLQYIEFGDSST-------------

>plant-like_FADS_Vitis_vinifera 312 bp
TVGG---IVALGKFDALHI--GHR-ELAIQASRVGAPFLLSFVGMAEVLGWE--------
--------RAPIVAKCDRKRVLSSWAPLC-GNVTPLEFLIEFSSVRHLTPRQFVEK-LYK
ELGVR-GVVAGENYRFGYKAAGDSSELVRLCEEF--GMGAYIIKPVMDNNQDSNS-K-ER
GQVSSTR--VRHALS-IGDMKYVSELLGRQHRLY-----TSSKHKVSAPKSCL-LP--PK
EGLYENCSLL-SCRVVIDTT--EVGPYK---SQ--GPWQVEFGV----------------

>plant-like_FADS_Prunus_persica 312 bp
--------VALGKFDALHI--GHR-ELAIQASKVGPPFLLSFVGMAEVLGWE--------
--------RAPIVARCDRKRVLSSWAPYC-GNMAPTEFQIEFSSVRHLTPRQFVEK-LLK
ELGVR-GVVAGENYRFGYKAAGDATELVRLCEEY--GIGAYIINSVMDKNDY-SDVK-DR
GQVSSTR--VRRALA-VGDMKYVSQLLGRQHRLKEQEGF-CCKNKLSVPKSCL-LG--PK
EGLYEKCYVFLSCNVVIDTE--EVGAS----TQ--DLSLIEFG-----------------

>plant-like_FADS_Carica_papaya 312 bp
VAGG---IVALGKFDALHV--GHR-ELAIQASKVGAPYLLSFVGMAEVLGWE--------
--------RAPVVAKCDRKRVLSSWAPYS--NIAPAEFEIEFSTVRHLAPRQFVEK-LSK
ELRVS-GVVAGENYRFGYKAAGDSSELVRLCQEY--GIGAYIINSVMDKNQDSDL-K-ER
GQVSSTR--VRRALA-VGDMDYVSELLGRPHRLD--H--SSGKFRMSVPKSYL-LP--PR
EGFYDHCCLL-KCRVLIDTT--EVICN----SQ--DYWFIEFGKLS--------------

>plant-like_FADS_Cucumis_sativus 312 bp
--------------------------------MVGSPFLLSFVGIAEVLGWE--------
--------RAPIVAQCDRQRVLSSWAPYC-QNSAPSEYRIQFSSVRYLTPREFVEK-LSK

```
ELRVC-GVVAGESYRFGYKAAGDAAELVKLCEEY--GISAYIIKSVMDRNQK-ANSK-ER
GQVSSTR--VRHALS-KGDMKYVSELLGRRHRLGLEGFS-NSNNRVSAPRSCL-LA--PK
EGLYNNCFVCIPCRVAIDST--DIGTSH---TQ--DRVNVEFGDEVV-------------
------------
>plant-like_FADS_Vaccinium_corymbosum 312 bp
---------------------GHR-ELAIQAAKVGVPFLLSFVGMAEVLGWE--------
--------RAPIVAKCDRKRVLSSWAPYC-GDTIPVEYELEFSRVRHLTPRQFVEM-LSK
KLGVR-GVVAGENYRFGYKAAGDSSELVKLSEEY--GMGAYIINTVMDKNQD-SNLK-EQ
GQVSSTR--VRLALA-KGDMKYVSELLGRQHRLDQEWV-ISNQHRVSARRSCF-LP--PK
EGLYESCSVVMPCKVTIDAS--DAATCI---SQ--DFQCIDFG-----------------
------------
>plant-like_FADS_Populus_trichocarpa 312 bp
VAGG---IVALGKFDALHI--GHR-ELAIQASKVGAPSLLSFVGMAEILGWE--------
--------RAPIVAKCDRSRVLSSWAPYC-GNLVPEEVQIEFSCVRHLTPRQFVEK-LSK
ELGVI-GVVAGENYRFGYKAAGDASELVRLCKEH--GMGAYIISSVMDNNQDYDL-K-DR
GQVSSTR--VRQALA-VGDMKYVSELLGRHHRLDQEE--SSG-WRVSAPKSYL-LP--PK
DGFYENCSLL-TCRVFMDTT--EADPFD---DQ--EPHLIEFGDSRPDRD----------
------------
>plant-like_FADS_Oryza_sativa 312 bp
VIDG---IVALGKFDALHI--GHR-ELAMYASKAGTPFLLSFVGIAEVLGWE--------
--------RPPIVAQCDRKRVLTSWAPYC-KNVVPIEYQVEFSKVRYLTPRQFVER-LSR
DLKIQ-GVVAGENYRFGYRASGDAAELVKLCEEF--GLSAFIVRSVMDTAR-SVNSS-DK
GQVSSSR--VRHALA-MGDMEYVSELLGRKHRL------LQERKRIMLPKSCM-MP--PA
DGLYENCDLL--CRVIINSE--ENSLL----IQ--ENQQIEFG-----------------
------------
>plant-like_FADS_Malus_x_domestica 312 bp
VAGG---IVALGKFDALHI--GHR-ELAIQASKVGPPFLLSFVGMAEVLGWE--------
--------RAPIVAKCDRKRVLSSWAPYC-GDMAPAEFQIEFSSVRCLTPRQFVEK-LVK
ELGVR-GVVTGENYRFGYKAAGDATELVRLCEEY--GIEAYIINSVMDKNEY-SDLKSDR
GQVSSTR--VRRALA-VGDMKYVSQLLGRQHRLKDQEGF-GCKHKLSVPKSSL-LA--PK
EGLYEKCYLCSSCRVVVDTG--EVDVCV---TH--DLRLIEFG-----------------
------------
>plant-like_FADS_Glycine_max 312 bp
VAGG---IVALGKFDALHI--GHR-ELAIQASRAGPPFLLSFVGMAKVLGWE--------
--------RAPIVAKCDRKRILSSWVPYC-CNMVPEEFEVEFSSVRHLNPRQFVEK-LSK
ELRVR-GVVAGENYRFGYKAAGDALELVKLCEEY--GMEAYIIKSVMDKN-RFTNSK-ER
GQVSSTR--VREALA-VGDLKYVSELLGRPHRLDQERFSGQYK--VSAPRSCL-LA--PK
EGLYEKCS--VQCSVVIDSK--YGGLSD---SQ--NLKFIEFG-----------------
------------
>plant-like_FADS_Solanum_tuberosum 312 bp
LTGG---IVALGKFDALHI--GHA-SLRSSSXER-IXVLLSIVGNAGSYLNG--------
-------GLLNVIAKG-SFRLGLLIAVMCQGNS--RI---DFSKVRYLTPCQFVEK-LSK
ELGVR-GVVAGENYRFGYRAAGDASDLVKLCEEY--GLEAYIINSVMDTNQISGKRE-QG
GQVSSTR--VRYALH-KGDMKYVSELLGRNHRLDQERF-TSERNRLSAPKSCL-LA--PK
EGLYENCSVLIPCRVIVDTT--EVDKFS---SQ--DLKIIDFG-----------------
------------
>plant-like_FADS_Ostreococcus_lucimarinus 312 bp
ATGERPLAVCLGKFDAMQP--GHL-ALAERAATRGDVVVVSXSGMAETLGWE--------
--------RLPITAPSDRSRVLAMWSGTT-GAAVSEH-ALAFGDVRELSPEAFVEM-LAR
-MGVG-AVVTGKNYRFGYKAAGDVEALKRFGEAN--GLTVDVVDLV-------AGL---G
DQVSSSR--IRSALS-AGDIDEVNEMLGREHRVG-ERAADRNLDEINLSLESA-QP--PS
DGAYSA------------------------------------------------------
------------
>plant-like_FADS_Ananas_comosus 312 bp
------------KFDALHI--GHR-QLAIHASKAGTPFLLSFIGMAEVLGWE--------
--------RPPIVAKCDRKRVLSSWAPYC-GNVAPLEYQVEFSKVRYLTPRQFVER-LSK
ELRIS-GVVAGENYRFGYKASGDASELVRLCKEY--GLSAFIVSPVMDKANRSINSS-DK
GQVSSTR--VHHALA-TGDIDYVTELLGRKHRL------LSMEKRILAPRSCM-QP--PG
DGMYENCDFP--CRVAINAE--PSFWQE---TK--DGDIIEFG-----------------
------------
```

>plant-like_FADS_Micromonas_sp_RCC299 312 bp
EDGGK-IVVALGKFDAMHR--GHA-ELARRASKMGAPILMSFGGMAEVLGWE--------
--------KLPVVAPGDRARVLEMWKAAC-EGKTVREHVIPFADIRRMSPEEFVST-L-K
DIGVG-GVVAGANYRFGFKAAGTADILKDLGEKL--GVDVSIVDLL-------AGYEN-T
PQVSSTR--VRACLA-AGDVEQAARLLGRNHALG-----AGNDGR--FPTSAA-QY--PA
IGRYAAIV-----RTPTTAE--EG-WGE---GN--DNVCVEFVGPAADA-----------

>plant-like_FADS_Triticum_aestivum 312 bp
VLDG---IVALGKFDALHI--GHR-ELAMHASKSGNPFLLSFVGMAEVLGWE--------
--------RPPIVAHCDRKRVLSSWAPYC-RNVVPLEYQVEFSKVRYLTPRQFVER-LST
DLRIK-GVVAGENYRFGYKASGDAAELVKLCEEF--GLSAFIVRSVMDTAKGSVNSS-DK
GQVSSSR--VRQALS-VGDIEYVSKLLGRKHRL------IKERKNIIVPKSCM-MP--PA
DGFYENCELF--CRVXINTE--ESSLS----FQ--EGKQIEFG-----------------

>plant-like_FADS_Picea_sitchensis 312 bp
VLGG---IVALGKFDALHV--GHR-ELAIRAAKIGVPFLLSFVGIAEVLGLE--------
--------RLPVVAKCDRKRVLSLWAPLC-DGVVPHEYHVQFAHVRYLSPRQFVEK-LSK
ELGVK-GVVAGANYRFGYKALGDASDLVQLCGEY--GLKAYIVDSVMDKFDGSGTDLREK
GQVSSTL--VRKALA-AGNIKRVEQLLGRKHRLN-----IVTKNIFVSCRSSV-QP--PR
EGQYG-CMIMDVDRNSNGNEQIEVTLHE---AHL-NGKSLEFEGSREC------------

>plant-like_FADS_Mimulus_guttatus 312 bp
VAGG---IVALGKFDALHI--GHR-ELAIQAAKIGVPFLLSFVGMAEILGWE--------
--------RAPIVAKCDRKRVLSSWAPLC-GNIVPKEFQIDFSKVRSLSPQQFVEK-LAE
ELGVA-GVVAGQNYRFGYRAAGDASDLVRLCNEY--GMRASIINSVMDNNQES-NSH-EK
GQVSSTR--VRHALA-KGDMKYVTELLGRHHRLNSENVFERKRLLV--PRSCL-LA--PK
EGLYENCSLVVPCRLVIDTT--ELAPPI---TQY-FESLIEFSDSKV-------------

>plant-like_FADS_Gossypium_raimondii 312 bp
VAGR---IVALGKFDALHI--GHR-ELAIQASKVGTPYLLSFVGMAEVLGWD--------
--------RPPVVAQCDRERVLSSWAPYC-GNVAPKEFQVQFMSVRHLSPRQFVEK-LAR
ELGVC-GVVAGENYRFGYKAAGDASELVRLCDEF--GMGAYIINSVMDRHQDALDLK-DR
GQVSSTR--VRQALA-EGDMKYVSELLGRPHRLDWESLTTSSTQRMSAPRSSL-LP--PK
DGFYENC---VTCRVSIDTS--RVDFCD---SQ--KSQVIEFG-----------------

>plant-like_FADS_Brachypodium_distachyon 312 bp
ISDG---IVALGKFDALHI--GHR-ELATHASRSGNPFLLSFVGMAEVLGWE--------
--------RPPIVARSDRERVLSSWAPYC-RNVVPLEYQVEFSKVRYLTPRQFVER-LST
DLRIK-GVVAGENYRFGYKASGDAAELVKLCEEF--GLSAFIVRSVMDTAKRSVNST-DK
GQVSSSR--VRHALS-VGDMEYVSKLLGRKHRL------LQEREKIVFPKSCM-MP--PA
DGFYENCGLV--CRVTINSE--KNSLT----FR--EDQQIEFG-----------------

>plant-like_FADS_Brassica_napus 312 bp
VSGG---IVALGKFDALHV--GHR-ELAIQASRVGTPYLLSFVGMAEVFGWE--------
--------RAPIVAKCDRKRVLTSWDSYC-GNKAPVEYEIEFASVRHLTPRQFVEK-LSK
ELRVC-GVVAGENYRFGYKASGDASELVRLCEEY--GIGAYIITSVMDKKQDSGDSK-DR
GQVSSTR--VRQALA-AGDMSYVSELLGRAHRLNTG---MQSERRISVPRSSV-LP--PG
NGVYNAC---VPCSVVVDSS--ELLLCN--------------------------------

>plant-like_FADS_Solanum_lycopersicum 312 bp
LTGG---IVALGKFDALHI--GHR-ELAIQAAKRGIPFLLSFVGMAEVLGWE--------
--------RAPIVAECDRKRILSSWAPYC-GNVMPKETQIDFSKVRYLTPCQFVEK-LSK
ELGVR-GVVAGENYRFGYRAAGDASDLVKLCEEY--GLEAYIINSVMDTNQISGK---ER
GQVSSTR--VRYALHSKGDMKYVSELLGRNHRLDQERF-TSERNRLSAPKSCL-LA--PK
EGLYENCSVLIPCRVIVDTT--EVASFS---SQ--DLKIIDFG-----------------

>plant-like_FADS_Chlamydomonas_reinhardtii 312 bp
------MGAVLGWPDRLPL--TAPQDRGRVLAGWAAALRSAASAVGSANGAT--------
--------RPSTEGNNGNGGTGTGAGDCG-AGRVVRLRTMPFGQIRGMSPEEFVAL-LAR
DLGAA-GVVAGRNYRFGFKAAGDAGALVRLGAQY--GLKVSIVDLVVAGASSSKAVA-ET

```
R-VSSSR--IRGLLE-GGHVEAVETLLGRKYRLAAAATAALGTGLLRVPAACY-QA--PA
PGQYRVMVRLAFMRTSLSST--EPVVGP---VR---ISELEVDLGPPGVHG---------
-SDSPPPRPLLL
>plant-like_FADS_Citrus_sinensi 312 bp
-----------------------------------------FIGMAEVFGWE--------
--------XAPIVAKCDRKRVLSSWAPYC-GNVAPVEFQIQFSSVRHLSPQQFVEK-LSR
ELGVR-GVVAGENYRFGYKAAGDASKLVRLCEEY--GMDACIINSVMDKHQD-NDSK-ER
GQVSSTR--VRQALA-MGDMKYVSELLGRQHRLDHDELITSNKHRVSVPKSCL-LP--PK
EGFYGNSVLLVKCKICIDAS--KVXFCN---SQ--EL-VIEFG-----------------
------------
>plant-like_FADS_Physcomitrella_patents 312 bp
VAGG---VVALGKFDALHV--GHR-ALAEHAAEIGAPFLVSFAGMAEVLGWE--------
--------RLPVVARCDRARVMSLWAEHC-GDVVPQEYMLEFSKVRSLSPEQFVEK-LAS
ELKVK-GVVAGANYRFGYKAAGDASDLVRLCQEY--GLQSAIVNPIMDASEMSSS-R-EK
GQVSTTR--VRKALA-NGDMKRVAELLGRRHRL------VRRDNLISVPAVNA-QP--PR
IGSYE-CSFVGNVRL-EES---QPSLVD---QQ--NLIVLDFLA----------------
------------
>plant-like_FADS_Ricinus_communis 312 bp
LAGG---IVALGKFDALHI--GHR-ELAIQASKVGTPYLLSFVGMAEVFGWE--------
--------RAPIVAKCDRNRVLASWAQYC-DNITPLEFQIQFSSVRHLNPQQFVEK-LSK
ELRVC-GVVAGENYRFGYKAAGDASDLVRLCEEY--GLGAYIINSVMDNKKDPDS-K-DR
GQVSSTR--VRQALA-LGDMKYVSKLLGRRHRLDQRG--SSSRWRISAPKSCL-LP--PK
DGFYKNCFLL-TCSAIIDSM--ELGLSI---SQ--DFQLVEFGD----------------
------------
>plant-like_FADS_Manihot_esculenta 312 bp
VAGG---IVALGKFDALHI--GHR-ELAIQASKVGSPYLLSFVGIAEVLGWE--------
--------RAPVVAKCDRSRVLTSWAPYC-GNIAPSEFHIEFSSVRHLSPQQFVEK-LSK
ELRVC-GVVAGENYRFGYKAAGDASELLRLCEEY--GMGAYIINSVMDKNKDSNDLK-DR
GQVSSTR--VRHALA-LGDMKYVSELLGRRHRLEEKGFTSSGRWKASIPKSCL-LP--PK
DGFYENC---VKCSVFIDST--EVGLNN---SQ--DFQLVEFGH----------------
------------
```

Figure S4.- HHPred alignment of structural template *Sy*NadM-Nudix (pdb 2qjo) and *At*RibF1.

```
>2qjo_A Bifunctional NMN adenylyltransferase/nudix hydrolase; two individual domains; HET: APR NAD; 2.60A
{Synechocystis SP}
Probab=98.10  E-value=6.8e-08  Score=62.13  Aligned_cols=239  Identities=12%  Similarity=0.076  Sum_probs=0.0

Q ss_pred             CCCCCCCCCEEEEECCCCCHHHHHHHHHHHHHCCCCEEEECCCCEEECCCCCCCCCCCCHHHHHHHHHH----CCCCEE
Q ss_conf             5578776738999667551478999999999857872575159962456799987047847899998755----699889
Q Wed_Jul_08_12:   77 EGLSPVAGGIVALGKFDALHIGHRELAIQAARIGTPYLLSFVGLAEVLGWKPRAPIVAKCDRKRVLSSWA----SYCGNI  152 (354)
Q Consensus       77 e~~~p~~G~VVaIG~FDGVHlGHQ~Li~~a~~~g~~~vltf~~~p~vl~~~p~~p~~~~~~r~r~l~~~~~----~~id~l  152 (354)
                     |.-.+-.--.+-.|.||-+|+||+.|+.++.+.....++-.     .-.+.++.+.-..+..+|+.+...    .+...+
T Consensus        1 ~~m~~k~~Igi~gGsFdP~H~GHl~l~~~a~~~~d~v~v~v-----~~~~~~~~~~~~~~~s~~~R~~Mi~~~~~~~~~~~~v   75 (341)
T 2qjo_A           1 DPMQTKYQYGIYIGRFQPFHLGHLRTLNLALEKAEQVIIIL-----GSHRVAADTRNPWRSPERMAMIEACLSPQILKRV   75 (341)
T ss_dssp             CCCSSSEEEEEEEECCTTCCHHHHHHHHHHHHHHEEEEEEEE-----EEETCCCCSSSCSCHHHHHHHHHHTTSCHHHHTTE
T ss_pred             CCCCCCCCEEEEEECCCCCHHHHHHHHHHHHHHCCCEEEEE-----ECCCCCCCCCCCCCHHHHHHHHHHHHHHCCCCEE
T ss_conf             987776418998046899878999999999998789279997-----07877877789989999999999864799779


Q ss_pred             EEEECCHHHHHHHCCCHHHHHHHHHHHHCCCEEEEEECCCCC--CCCCCCCCHHHHHHHHHHCCCEEEEEECCEEECCCCCCC
Q ss_conf             99844887864305999999999999875961999898545--577878899999999998497799964367657232 10
Q Wed_Jul_08_12:  153 APVEFEIEFASVRHLNPQQFVEKLSRELRVCGVVAGENYR--FGYRASGDASELVRLCKDFGISAYIINSVMDKNQVSVN  230 (354)
Q Consensus      153 ~~~~F~~eFa~i~~LSpeeFVe~Li~~L~~k~IVVG~DFr--FG~~r~Gdv~~L~~lg~~~G~~V~vV~~v~~~~~~~~~~  230 (354)
                     ........+.     +++..+..+....  ..++.|.|..  |++.+.-....++..   .++.+..++...+
T Consensus       76 ~~~~~~~~~~~~-----~~~~~~~~~~~~~~~--~~~i~~~~~~~~~~~~~~~~~~~~~~~~~---~~~~~~~~~~~~~~~-------  138 (341)
T 2qjo_A          76 HFLTVRDWLY-----SDNLWLAAVQQQV--LKITGGSNSVVVLGHRKDASSYYLNLF---PQWDYLETGHYPD-------  138 (341)
T ss_dssp             EEEEEECCTT-----CHHHHHHHHHHHH--HHHHTTCSCEEEEECCCSGGGGGGGSC---TTSEEEECCCCTT-------
T ss_pred             EEEECCCCCC-----CHHHHHHHHHHHC--CEEEECCCHHHHHHHHHHHHHHHHHHCC---CCEEEEECCCCC-------
T ss_conf             9982543356-----6177999999617--55784163266777787776455541 7---9758997788678-------


Q ss_pred             CCCCCCCCCCCCEEEHHHHHHHHHHCCCH-----------------------------------HHHHHHCCCCEEEEEEE
Q ss_conf             012444345883774899999999849 99-----------------------------------99999719972999999
Q Wed_Jul_08_12:  231 TEEEDSKSKERGQVSSTRVRHALAAGDV-----------------------------------RYVTELLGRPHRVISRT  275 (354)
Q Consensus      231 ~e~~d~~~~~d~~kVSSTrIR~lL~~Gdi-----------------------------------~~A~~LLGRpY~i~G~V  275 (354)
                                  ||||.||++|.+|+.                                   ....++-+.||.-....
T Consensus      139 -------------ISST~IR~~i~~g~~~~~~~~lVP~~V~~~I~~~~~~~~~~~~~~~~~~~e~~~l~~~~~~~~~~~~~~~~~~~~~~~~~  205 (341)
T 2qjo_A         139 -------------FSSTAIRGAYFEGKEGDYLDKVPPAIADYLQTFQKSERYIALCDEYQFLQAYKQAWATAPYAPTFIT  205 (341)
T ss_dssp             -------------CCHHHHHHHHHHTCGGGTTTTSCHHHHHHHHHHTTSHHHHHHHHHHHHHHHHHHHHHHTTTTSSSCCCEEE
T ss_pred             -------------CCHHHHHHHHHHCCCCCHHHHHHHHHHHHHHHHHHHHCCHHHHHHHHHHHHHHHHHHHHHHHHHCCCCCCCCCCEEE
T ss_conf             -------------676078999976996308887449999999999619198999999999999865654055157870378


Q ss_pred             EECCCCCCCCCCCCCCCHHHHHCCCCCEEEEE-EEEECCCCCCEEEEEE-------EECCCCCE---EEEEEEEEECCCCCC
Q ss_conf             9687456786347874664106898806999-999859520652665-------78897744---788766620457644
Q Wed_Jul_08_12:  276 RTQDLTSKRGRISLQTSSLLNLPPGNGVYKA-CSLIVGDKHPISCKV-------IVDTSNLY---IETEEERFHNSDESQ  344 (354)
Q Consensus      276 v~G~~~~~~Gr~IGfPTANl~~l~P~~GVYa~-~~~~i~G~~~~~~~~~~~~~~~-------~~~~~~~~~~~~---iEt~Ev~lfDFd~Di  344 (354)
                     +..=-...|+-|=.-.++-    |..|.|+- ...++.++.+.-|..       +++.....   .-..-...-+.+.+.
T Consensus      206 v~~iv~~~g~vLlv~r~~~----~~~g~w~lPgG~~e~gE~~~~~aa~REl~EEtGl~v~~~~~~~~~~~~~~~~~~~~~~~~~~~~~~~~  281 (341)
T 2qjo_A         206 TDAVVVQAGHVLMVRRQAK----PGLGLIALPGGFIKQNETLVEGMLRELKEETRLKVPLPVLRGSIVDSHVFDAPGRSL  281 (341)
T ss_dssp             EEEEEEEETTEEEEEEECCSS----SSTTCEECSEEECCTTSCHHHHHHHHHHHHHHHHCCSSCHHHHHHTEEEEEEECCTTSCT
T ss_pred             EEEEEEECCEEEEEEEECCC----CCCCCEECCCCCCCCCCCCHHHHHHHHHHHHHHHHCCCEECCEEEEEEEEEEEECCCCCCCC
T ss_conf             99999979999999844 89----8998186987727899799999999999979731111478898645641578 8887


Q ss_pred             CCEEEEEECC
Q ss_conf             7758999729
Q Wed_Jul_08_12:  345 EFQLLGIEFG  354 (354)
Q Consensus      345 ygq~l~VEF~  354 (354)
                     ..+.+.+-|-
T Consensus      282 ~~~~i~~~~~  291 (341)
T 2qjo_A         282 RGRTITHAYF  291 (341)
T ss_dssp             TSCEEEEEEE
T ss_pred             CCEEEEEEEE
T ss_conf             6149999999
```
